# Role of the *M* point phonons for the dynamical stability of B2 compounds

**DOI:** 10.1038/s41598-022-10658-2

**Published:** 2022-05-04

**Authors:** Shota Ono, Daigo Kobayashi

**Affiliations:** Department of Electrical, Electronic and Computer Engineering, Gifu University, Gifu, 501-1193 Japan

**Keywords:** Materials science, Physics

## Abstract

Although many binary compounds have the B2 (CsCl-type) structure in the thermodynamic phase diagram, an origin of the dynamical stability is not understood well. Here, we focus on 416 compounds in the B2 structure extracted from the Materials Project, and study the dynamical stability of those compounds from first principles. We demonstrate that the dynamical stability of the B2 compounds lies in whether the lowest frequency phonons around the *M* point in the Brillouin zone are endowed with a positive frequency, except for VRu. We show that the interatomic interactions up to the fourth nearest neighbor atoms are necessary for stabilizing such phonon modes, which should determine the minimum cutoff radius for constructing the interatomic potentials of binary compounds with guaranteed accuracy.

## Introduction

Since the characterization of the crystal structure of CsCl in 1921^1^, many of the B2 (called CsCl-type) compounds have been synthesized experimentally, resulting in that the B2 structure is the most common phase in the thermodynamic phase diagram of binary compounds. Analyses of materials database have shown how frequent they appear^2–4^. More recently, Kolli *et al*. identified 267 parent crystal structures that can generate their derivative ordered phases, and showed that the body-centered cubic (bcc) structure is the most common parent crystal structure^4^ by using the Materials Project (MP) database^5^. Among them, the B2 structure is the most common ordering on bcc structure. Apart from the ambient condition, the B2 structure may also appear: the B2 structure can be transformed from the B1 (NaCl-type) structure under high pressure (such as alkali halides and alkali-earth oxides^6,7^) and from the L1$_0$ (CuAu-type) structure in warm dense matter regime^8^.

Although the elastic stability of the B2 structure has been studied in a wide variety of compounds^9–11^, such a stability does not always yield the dynamical stability against the zone boundary phonon excitations^12^. For the parent bcc structure, it is well known that the transverse acoustic phonon at the *N* point in the Brillouin zone (BZ), propagating along the diagonal direction of any two axes in the cubic cell, has relatively low frequencies^13,14^. It has been shown that such a phonon is stabilized by long-range interatomic interactions, allowing alkali metals to form the bcc structure at the ambient condition^15^. By considering the fact that the B2 structure is equivalent to the bcc structure when two species are assumed to be the same element, we expect that a similar scenario holds: the lowest frequency phonon at the *M* point that is stabilized by the long-range interactions determines the dynamical stability of the B2 compounds. We have recently confirmed that the long-range interatomic interactions up to the fifth nearest neighbor (5NN) atoms are needed to understand the dynamical stability of the CuAu in the L1$_0$ structure^8^.

The range of the interatomic interactions is of prime importance in the field of atomistic modeling of condensed matters. For the bcc elemental metals, the cutoff radii should be larger than the 3NN or 4NN distances^16–18^. However, it has not been understood why more than 3NN distances are required to describe the potential energy surface accurately.

In this paper, we extract 416 compounds in the B2 structure from the MP^5^, and study the dynamical stability of the B2 compounds using density-functional theory (DFT) and density-functional perturbation theory (DFPT). By assuming zero temperature and pressure, we show that 266 out of 416 compounds are dynamically stable, and demonstrate that such a stability is mainly determined by whether the lowest frequency phonons around the *M* point in the BZ are endowed with a positive frequency, except for VRu. In addition, we develop a force constant model taking into account the interatomic interactions up to the 6NN atoms, and demonstrate that the interatomic interactions up to the 4NN atoms are enough to stabilize the lowest energy phonons at the *M* point. This should determine a minimum cutoff radius of the interatomic potentials for binary compounds. We also discuss to what extent the formation energy is correlated with the dynamical stability of B2 compounds. The present work unveils the microscopic mechanism of the dynamical stability for the B2 phase, which stimulates other studies involving different crystal structures and gives a useful insight for developing the interatomic potentials of binary systems.

The present work assumes the B2 ordering on bcc structure. A competition between the B2 ordering and the finite temperature effects play an important role to understand the stability of binary^19^ and high-entropy alloys^20^. Exploring a phonon stabilization under such situations is left for future work. In addition, the role of the spin-orbit coupling on the metastability needs to be investigated when the compound includes heavy elements^21^.

**Figure 1:**
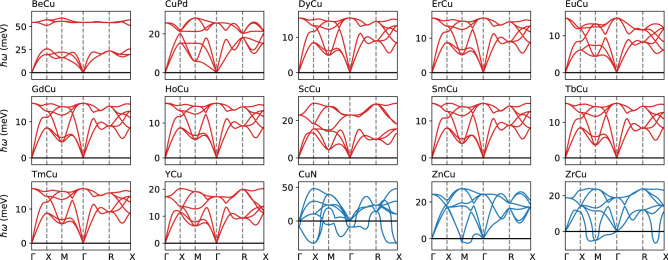
The phonon dispersions of the Cu-based B2 compounds. The dispersion curves are colored red and blue for the dynamically stable and unstable compounds, respectively.

**Figure 2:**
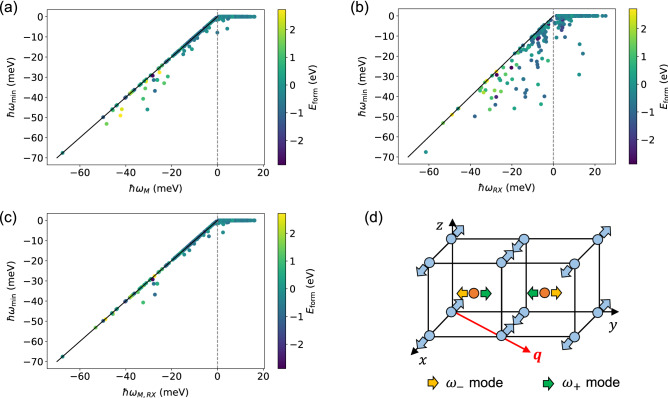
The minimum phonon energy plotted as (**a**) $\hslash \omega _j = \hslash \omega _M$, (**b**) $\hslash \omega _{RX}$, and (**c**) $\hslash \omega _{M, RX}$. The solid line indicates the relation of $\omega _{j}=\omega _\mathrm{min}$. The color of the data points indicates the value of the formation energy extracted from the MP database^5^. (**d**) The displacement vectors of the *M* point phonon modes having the frequencies of $\omega _{\pm }$ (doubly degenerated). The cases that the atoms *A* and *B* move along the *x* and *y* directions, respectively, are illustrated.

## Results and discussion

### Phonon dispersions

We have found that 266 out of 416 compounds are identified to be dynamically stable. The phonon dispersions for the 416 compounds are shown in the Supplementary Information. For example, we show the phonon dispersions for the 15 Cu-based compounds in Fig 1. The 12 compounds are dynamically stable, whereas the others (CuN, ZnCu, and ZrCu) are unstable. Although the former 12 compounds are stable, the phonon energies of the transverse acoustic branch at the *M* point and around the middle of the *R*-*X* line tend to be small. In a similar manner, the instabilities of the CuN, ZnCu, and ZrCu are due to the phonon softening around these points, leading to negative phonon energies. The CuN also shows strong phonon softening at the *X* point.

For the B2 compounds including the platinum-group metals (Os, Ru, Ir, Rh, Pt, and Pd), Hart *et al*. have identified that 16 compounds have the B2 structure as the ground state by using the DFT calculations within the generalized gradient approximation (GGA) of Perdew-Burke-Ernzerhof (PBE)^22^. We have also identified that the 16 compounds are dynamically stable. In addition, we find that the B2 FeRh is dynamically stable, which is consistent with the experimental synthesis^23^, whereas the FeRh in the B2 structure has been predicted to be unstable^22^.

### The *M* point phonons

In order to understand the dynamical stability of the B2 compounds, we plot the minimum phonon energy within the entire BZ ($\hslash \omega _\mathrm{min}$) as a function of the lowest phonon energy around the *M* point ($\hslash \omega _{M}$) in Fig. 2(a), where the $\omega _{M}$ was determined by searching the minimum value of the phonon energy from the middle of the *X*-*M* line to the *M* point to the middle of the *M*-$\Gamma$ line. The $\omega _{M}$ is strongly correlated with the $\omega _\mathrm{min}$: when $\omega _{M}< 0$, a linear relationship of $\omega _{M}=\omega _\mathrm{min}$ almost holds, whereas when $\omega _{M}>0$, an equality of $\omega _\mathrm{min}=0$ holds except for some compounds. We also investigated the $\hslash \omega _\mathrm{min}$ as a function of the lowest phonon energy along the *R*-*X* line ($\hslash \omega _{RX}$), as shown in Fig. 2(b). While the $\omega _{RX}$ is weakly correlated with the $\omega _\mathrm{min}$, no linear relationship is observed for $\omega _{RX}<0$. Figure 2(c) shows the $\omega _\mathrm{min}$ versus the minimum value of $\omega _{M, RX} = \min (\omega _{M}, \omega _{RX})$, which improves the linear relationship for $\omega _{M, RX}<0$, compared to Fig. 2(a). These plots show that the dynamical stability of the B2 compounds is correlated with the phonon energies around the *M* point and along the *R*-*X* line. In particular, the impact of the phonon dispersions around the *M* point is significant.

We next discuss some exceptions that do not follow the dynamical stability criteria, that is, if $\omega _{M}>0$, then $\omega _\mathrm{min} = 0$. In Figs. 2(a) and 2(c), there are 5 compounds (LuMg, PrMg, VRu, YMg, and ZrRh) that satisfy both $\omega _{M}, \omega _{M, RX}>0$ and $\hslash \omega _\mathrm{min}<\varepsilon _\mathrm{min}$, where $\varepsilon _\mathrm{min}$ is set to be $-1$ meV in the present work. For the cases of LuMg, PrMg, and YMg, the minimum phonon energies appear around the $\Gamma$ point, while for the VRu and ZrRh, they appear along the $\Gamma$-*R* line (see the Supplementary Information). The values of $\hslash \omega _\mathrm{min}$ are less than $\varepsilon _\mathrm{min}$ but larger than $2\varepsilon _\mathrm{min}$, so that their instability may be due to a finite size of the *q* grid used in the phonon dispersion calculations. However, the instability of VRu is anomalous because $\hslash \omega _\mathrm{min}=-6$ meV is observed only along the $\Gamma$-*R* line in the BZ. We also used the local-density approximation (LDA) of Perdew-Zunger (PZ)^24^ for the exchange-correlation functional, and found that the phonon dispersions were similar to those within the GGA-PBE^25^ (see the Supplementary Information). It is noteworthy that the experimental synthesis of VRu has been reported by several groups^26,27^, where the B2 and L1$_0$ phases coexist at 110 K, while only the B2 phase survives at 360 K^27^. More recently, the incommensurate structure with complex geometry has been observed^28^. The tetragonal distortion observed in the L1$_0$ structure was estimated to be $c/a\simeq 1.06$^27^ and 1.07^28^, while the geometry optimization within GGA-PBE yielded $c/a=1.003 \ll 1.07$: the L1$_0$ structure was relaxed into the B2 structure. When zero temperature condition and a primitive unit cell containing two atoms are assumed, it will be difficult to rationalize the experimental synthesis of the VRu in the B2 and L1$_0$ structures. More investigation is left for future work.

We finally list unstable compounds with $\omega _{M}\ne \omega _\mathrm{min}$. We found that 11 compounds (including BrN, CuN, IN, InSb, IrN, MnS, NCl, PBr, PdN, PdO, and TcB) satisfy the equality of $\omega _{RX}=\omega _\mathrm{min}$ rather than $\omega _{M}=\omega _\mathrm{min}$. This was identified by comparing Fig. 2(a) with 2(c) for $\omega _{M}<0$ and $\omega _{M, RX}<0$, respectively. We also found that 9 compounds (including LaN, NbRu, NdN, PrN, ReN, TaRu, TcN, TiRh, and VRu) show neither $\omega _{RX} = \omega _\mathrm{min}$ nor $\omega _{M} = \omega _\mathrm{min}$, as shown in Fig. 2(c). As discussed, only VRu shows $\omega _{M}>0$.

The formation energy $E_\mathrm{form}$ may be another descriptor for understanding the stability of the B2 compounds: the dynamically stable compounds have negative $E_\mathrm{form}$ and, in turn, the unstable compounds have positive $E_\mathrm{form}$. However, many exceptions have been found: (i) the six compounds of CaNi (0.024), CeMg (0.087), CrCo (0.154), LiBe (0.366), MnZn (0.062), and YbRu (0.209) have positive $E_\mathrm{form}$ (eV/atom), where the figure in a parenthesis indicates the magnitude of $E_\mathrm{form}$, although they are dynamically stable. In contrast, (ii) 103 out of 150 unstable compounds have negative $E_\mathrm{form}$: for example, CsF ($-2.734$), LiF ($-2.878$), RbF ($-2.775$), and SrO ($-2.662$) for strongly bonded systems and AlRe ($-0.010$), CrN ($-0.013$), and MnAu ($-0.010$) for weakly bonded systems. A similar issue has been found in a wide variety of materials, such as ordered alloys^29,30^ and many two-dimensional materials^31–33^. The metastability of materials has to be studied in detail.

It is noteworthy that the B2 compounds including a semiconducting element (group 14-17) tend to be unstable (see Table S1 and Fig. S1 in the Supplementary Information). Such compounds include the strongly bonded systems mentioned above. The instability of these may be due to the presence of different ground state structure such as the B1 structure. The understanding for the group dependence of the dynamical stability is left for future work.

**Figure 3:**
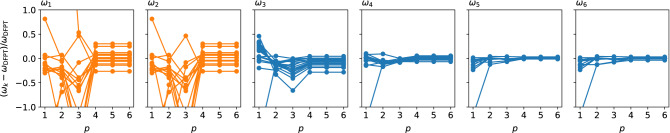
The variation of the relative error of the *M* point phonon energies between the *p*NN models and the DFPT for the 15 Cu-based compounds.

### Effect of long-range interatomic interactions

We next study an origin of the positive value for the phonon energies at the *M* point and identify the role of the long-range interatomic interactions. Based on standard lattice dynamics^34^, we derived analytical expressions for the phonon frequencies for the wavevector $\varvec{q}=(\pi /a,\pi /a,0)$, at the *M* point in the BZ, by assuming the B2 compounds that consist of atoms $\kappa =A$ and *B* with the masses $M_\kappa$. By using the translational symmetry of the crystal, the dynamical matrix $\tilde{D}$ is given by

1 $$\begin{aligned} \tilde{D}_{\alpha \beta }^{\kappa \kappa '}(\varvec{q}) = \frac{1}{\sqrt{M_\kappa M_{\kappa '}}} \sum _{j} D_{\alpha \beta }^{\kappa \kappa '}(\varvec{R}_j,\varvec{0}) e^{-i\varvec{q}\cdot \varvec{R}_j}, \end{aligned}$$

where $D_{\alpha \beta }^{\kappa \kappa '}(\varvec{R}_j,\varvec{0})$ is the force constant matrix, i.e., the force along the direction of $\alpha$ acting on the atom $\kappa$ in a unit cell characterized by the lattice vector $\varvec{R}_j$ when the atom $\kappa '$ in the cell of $\varvec{R}=\varvec{0}$ is displaced along the direction of $\beta$. With the *q* grid used in the present work, the $\varvec{R}_j$ can take the vectors of $\sum _{i=1}^{3} m_i\varvec{a}_i$ with $m_i = -1,0,1,2$, where $\varvec{a}_i$’s are the primitive lattice vectors in the cubic cell. We considered the force constants up to the 6NN sites of B2 *AB*. By assuming that the atom *A* is located at the origin, the position of the first, second, third, fourth, fifth, and sixth NNs are *B*(*a*/2, *a*/2, *a*/2), *A*(*a*, 0, 0), *A*(*a*, *a*, 0), *B*(3*a*/2, *a*/2, *a*/2), *A*(*a*, *a*, *a*), and *A*(2*a*, 0, 0), respectively, and these equivalent sites, where the numbers of the equivalent sites are 8, 6, 12, 24, 8, and 6, respectively. Due to the equivalence between the *x* and *y* directions, four different phonon modes are present: the *z*-polarized modes having $\omega _{z\kappa }^{2}= \tilde{D}_{zz}^{\kappa \kappa }(\varvec{q})$ ($\kappa =$ A or B) and the *x*- and *y*-polarized modes having

2 $$\begin{aligned} \omega _{\pm }^{2}= \frac{1}{2}\left[ \tilde{D}_{xx}^{AA}(\varvec{q})+\tilde{D}_{yy}^{BB}(\varvec{q}) \pm \tilde{C}_{xy}(\varvec{q}) \right] , \end{aligned}$$

with

3 $$\begin{aligned} \tilde{C}_{\alpha \beta }(\varvec{q})= & {} \sqrt{\left[ \tilde{D}_{\alpha \alpha }^{AA}(\varvec{q}) -\tilde{D}_{\beta \beta }^{BB}(\varvec{q})\right] ^2 +\left[ 2\tilde{D}_{\alpha \beta }^{AB}(\varvec{q})\right] ^2}. \end{aligned}$$

The value of $\omega _{\pm }^{2}$s in Eq. (2) does not change when the indexes *x* and *y* are replaced with *y* and *x*, respectively, leading to the doubly degenerated modes. The displacement patterns of the normal modes with $\omega _{\pm }$ are described by a combination of the $x \ (y)$-polarized vibration of the atom *A* and the $y \ (x)$-polarized vibration of the atom *B*, which are shown in Fig. 2(d). From the expressions of Eqs. (2) and (3), one can expect that the coupling term of $\tilde{D}_{\alpha \beta }^{AB}(\varvec{q})$ plays an important role to yield the positive value of $\omega _{-}^{2}$. The derivations of these expressions are provided in the Supplementary Information.

To study how the *M* point phonons are stabilized by the interatomic interactions, we introduce the *p*NN model taking into account the force constants up to the *p*th NN atoms and compare the *M* point phonon energies with the DFPT results. The errors of the phonon energies with restricted interactions, with respect to the DFPT results, are plotted as a function of *p* in Fig. 3. For clarity, the cases of the 15 Cu-based compounds are shown. The phonon frequencies are denoted as $\omega _k$ with $k=1,\cdots , 6$ in an ascending order. The lowest energy phonons ($\omega _1$ and $\omega _2$) correspond to the normal modes having the frequency $\omega _{-}$ except for CuN. For the low energy phonon modes, the deviation from the DFPT is large when $p\le 3$. The $p=4$ is a critical value for the convergence of the phonon energy, and such a *p* corresponds to the interatomic interactions between different atoms *A* and *B*, which is consistent with our expectation above. On the other hand, $p=2$ is found to suffice for the high energy phonons. These results indicate that the low and high energy phonons at the *M* point are stabilized by the long-range and short-range interatomic interactions, respectively. The instability of the *M* point phonon modes in the CuN was due to the negative value of $\omega _{z\kappa }^{2}= \tilde{D}_{zz}^{\kappa \kappa }(\varvec{q})$ with $\kappa =$ Cu.

The interatomic distance with the $p=4$ atoms is $\sqrt{11}a/2\simeq 1.66a$, and the total number of the NN atoms up to $p=4$ is 50. This might be a minimum criteria for determining the cutoff radius $(R_c)$ of the interatomic potentials in the *AB*-type compounds with guaranteed accuracy. Interestingly, Seko *et al.* have constructed an interatomic potential for the bcc K with $a=5.284$ Å, and shown that the energy, force, and stress calculated by using the interatomic potential agree well with those obtained by the DFT calculations when the value of $R_c$ is set to be more than 9 Å^17^. Such a $R_c$ is quite similar to $\sqrt{11}a/2=8.762$ Å.

Some compounds have a large discrepancy of the lowest phonon energy between the 6NN model and the DFPT. For example, the CdAg, MnHg, ZnCu, ZnAg, and ZnAu systems have $(\omega _{1},\omega _\mathrm{DFPT})=(-3.8,-0.9)$, $(-2.0,1.5)$, $(-5.7,-1.7)$, $(-3.5,3.0)$, and $(-3.2,2.4)$, respectively, in units of meV. This implies that more long-range interactions are required to achieve the convergence to the DFPT results. It should be noted that the stability of the *d* electron compounds might not be described accurately within the PBE approximation. The value of $E_\mathrm{form}$ tends to be overestimated within the PBE when one studies the B2 compound that consists of the atoms having the completely filled *d* orbitals such as Cu, Ag, Au, Zn, and Cd^35^. More analysis using other functionals is beyond the scope of this work. The comparisons between the *p*NN and the DFPT for the 416 compounds are provided in the Supplementary Information.

## Methods

By using the MP^5^ and the pymatgen^36^, we first extracted the list of the B2 compounds. By setting the space group to $Pm\bar{3}m$ with the number of atoms in a unit cell being two and excluding the atoms in the actinide series, 416 B2 compounds having the inorganic crystal structure database (ICSD) IDs were found.

We next optimized the lattice parameter *a* for the 416 compounds. All the DFT calculations were performed with the Quantum ESPRESSO (QE)^37^ using the ultrasoft pseudopotentials provided in the pslibrary.1.0.0^38^. We used the GGA-PBE^25^ functional for the exchange-correlation energy, unless noted otherwise. Spin-polarized approximation was used for all calculations. The cutoff energies for the wavefunction and the charge density are 60 Ry and 600 Ry, respectively, and 20$\times$20$\times$20 *k* grid was used in the self-consistent field (SCF) calculations^39^. The SCF convergence threshold was set to be $10^{-8}$ Ry and the smearing parameter of $\sigma =0.02$ Ry^40^ was used for all calculations. The total energy and forces were converged within $10^{-5}$ Ry and $10^{-4}$ a.u., respectively.

The accuracy of the present calculations was checked by comparing the optimized *a*’s with those in the MP^5^. We have confirmed that the optimized *a*’s agree with the reference values within an error of 1 % except for the 11 Ce-based compounds, ClO, and NCl. The optimized *a*’s in the Ce-based compounds are larger than the reference values by a few percent. This may be due to the absence of the *f*-electrons in the present calculations, resulting in no magnetic moments, whereas the Ce-based compounds show ferromagnetic phase in the MP^5^. The error of the *a*’s for ClO and NCl were 4.6 and 11.1 %, respectively.

Although the formation energies $E_\mathrm{form}$ of the 416 compounds can be obtained from the MP^5^, the dynamical stability properties are not always obtained. We thus performed phonon dispersion calculations based on DFPT^41^ implemented in QE. The threshold parameter for the self-consistency (tr2_ph) was set to be $10^{-14}$, and 4$\times$4$\times$4 *q* grid (10 *q* points) was used. We calculated the phonon dispersions along the symmetry lines $\Gamma$-*X*-*M*-$\Gamma$-*R*-*X*. When the phonon frequency $\omega$ is imaginary, the phonon energy is represented as a negative value, $-\hslash \vert \omega \vert$, with the Planck constant $\hslash$. In the present work that adapts a finite size *q* grid, we identify the B2 compound as dynamically stable if the lowest phonon energy is larger than $\varepsilon _\mathrm{min}=-1$ meV.

## Supplementary Information

Supplementary Information 1.

Supplementary Information 2.

## Data Availability

The data that support the findings of this study are available from the corresponding author upon reasonable request. The corresponding author, on behalf of all authors of the paper, is responsible for submitting a competing interests statement.
