# Supplementary material for: Role of the M point phonons for the dynamical stability of B2 compounds
Source: Sci Rep. 2022 May 4;12:7258. doi: 10.1038/s41598-022-10658-2 (PMC9068927; doi:10.1038/s41598-022-10658-2)
Supplement: Supplementary file 1 — Supplementary Information 1. [file 41598_2022_10658_MOESM1_ESM.pdf]

Supplementary Information for "Role of the M point phonons for the dynamical stability of B2 compounds"

Shota Ono* and Daigo Kobayashi
Department of Electrical, Electronic and Computer Engineering, Gifu University, Gifu 501-1193, Japan

We provide (i) the analytical expressions for the phonon energies at the M point, $(\pi/a, \pi/a, 0)$, assuming the B2 compounds AB, (ii) the list of the 416 B2 compounds AB (see Table S1), (iii) the phonon dispersions for the B2 AB (see Fig. S1), and (iv) the phonon dispersions for the VRu (see Fig. S2). We also provide (v) the M point phonon energies calculated within the pNN models and the DFPT (see the pNN directory).

Based on standard lattice dynamics [31], the atomic motion in a crystal is described by

$$M_\kappa \frac{d^2 u_{\kappa\alpha}(\boldsymbol{R}_i)}{dt^2} = -\sum_{\kappa'\beta j} D_{\alpha\beta}^{\kappa\kappa'}(\boldsymbol{R}_i, \boldsymbol{R}_j) u_{\kappa'\beta}(\boldsymbol{R}_j), \quad \text{(S1)}$$

where $u_{\kappa\alpha}(\boldsymbol{R}_i)$ is the displacement components for the atom κ ($= A$ or B) along the direction of α ($= x, y, z$) in the unit cell characterized by the lattice vector $\boldsymbol{R}_i = (R_{ix}, R_{iy}, R_{iz})$ that is a linear combination of the primitive lattice vectors of $\boldsymbol{a}_1 = (a, 0, 0), \boldsymbol{a}_2 = (0, a, 0)$, and $\boldsymbol{a}_3 = (0, 0, a)$. M_κ is the mass of the atom κ and $D_{\alpha\beta}^{\kappa\kappa'}(\boldsymbol{R}_i, \boldsymbol{R}_j)$ is the force constant matrix defined as

$$D_{\alpha\beta}^{\kappa\kappa'}(\boldsymbol{R}_i, \boldsymbol{R}_j) = \frac{\partial^2 V}{\partial X_{\kappa\alpha}(\boldsymbol{R}_i) \partial X_{\kappa'\beta}(\boldsymbol{R}_j)}\Big|_0, \quad \text{(S2)}$$

where $X_{\kappa\alpha}(\boldsymbol{R}_i) = R_{i\alpha} + \tau_{\kappa\alpha}$ and $\tau_{\kappa\alpha}$ is the α-component of the basis vector for the atom κ: $(\tau_{Ax}, \tau_{Ay}, \tau_{Az}) = (0, 0, 0)$ and $(\tau_{Bx}, \tau_{By}, \tau_{Bz}) = (a/2, a/2, a/2)$. Assuming the plane wave solution with the wavevector $\boldsymbol{q}$, one obtains the eigenvalue equation

$$\omega^2 \epsilon_{\kappa\alpha}(\boldsymbol{q}) = \sum_{\kappa'\beta} \tilde{D}_{\alpha\beta}^{\kappa\kappa'}(\boldsymbol{q}) \epsilon_{\kappa'\beta}(\boldsymbol{q}), \quad \text{(S3)}$$

where $\epsilon_{\kappa\alpha}$ is the α-component of the polarization vector of the atom κ and, using the translational symmetry of the crystal, the dynamical matrix $\tilde{D}$ is given by (the same as Eq. (1))

$$\tilde{D}_{\alpha\beta}^{\kappa\kappa'}(\boldsymbol{q}) = \frac{1}{\sqrt{M_\kappa M_{\kappa'}}} \sum_j D_{\alpha\beta}^{\kappa\kappa'}(\boldsymbol{R}_j) e^{-i\boldsymbol{q}\cdot\boldsymbol{R}_j}, \quad \text{(S4)}$$

where we used the notation of $D_{\alpha\beta}^{\kappa\kappa'}(\boldsymbol{R}_j) = D_{\alpha\beta}^{\kappa\kappa'}(\boldsymbol{R}_j, \boldsymbol{0})$. Also, due to the translational symmetry, the acoustic sum rule holds: $\sum_{\kappa' j} D_{\beta\alpha}^{\kappa'\kappa}(\boldsymbol{R}_j) = 0$.

We consider the force constants up to the 6NN sites of B2 AB, as described in the main text. The 6×6 dynamical matrix is expressed as

$$\tilde{D}(\boldsymbol{q}) = \begin{pmatrix} \tilde{D}^{AA}(\boldsymbol{q}) & \tilde{D}^{AB}(\boldsymbol{q}) \\ \tilde{D}^{BA}(\boldsymbol{q}) & \tilde{D}^{BB}(\boldsymbol{q}) \end{pmatrix}, \quad \text{(S5)}$$

where the 3×3 diagonal matrix is given by

$$\tilde{D}^{\kappa\kappa}(\boldsymbol{q}) = \begin{pmatrix} \tilde{D}_{xx}^{\kappa\kappa}(\boldsymbol{q}) & 0 & 0 \\ 0 & \tilde{D}_{yy}^{\kappa\kappa}(\boldsymbol{q}) & 0 \\ 0 & 0 & \tilde{D}_{zz}^{\kappa\kappa}(\boldsymbol{q}) \end{pmatrix} \quad \text{(S6)}$$

and the 3×3 off-diagonal matrix for $\kappa \neq \kappa'$ is given by

$$\tilde{D}^{\kappa\kappa'}(\boldsymbol{q}) = \begin{pmatrix} 0 & \tilde{D}_{xy}^{\kappa\kappa'}(\boldsymbol{q}) & 0 \\ \tilde{D}_{yx}^{\kappa\kappa'}(\boldsymbol{q}) & 0 & 0 \\ 0 & 0 & 0 \end{pmatrix}. \quad \text{(S7)}$$

By solving Eq. (S3), one obtains the six phonon energies (the same as Eqs. (2) and (3) in the main text): $\omega^2 = \tilde{D}_{zz}^{AA}(\boldsymbol{q})$, $\tilde{D}_{zz}^{BB}(\boldsymbol{q})$,

$$\frac{1}{2}\left[\tilde{D}_{xx}^{AA}(\boldsymbol{q}) + \tilde{D}_{yy}^{BB}(\boldsymbol{q}) \pm \tilde{C}_{xy}(\boldsymbol{q})\right],$$
$$\frac{1}{2}\left[\tilde{D}_{yy}^{AA}(\boldsymbol{q}) + \tilde{D}_{xx}^{BB}(\boldsymbol{q}) \pm \tilde{C}_{yx}(\boldsymbol{q})\right], \quad \text{(S8)}$$

with

$$\tilde{C}_{\alpha\beta}(\boldsymbol{q}) = \sqrt{\left[\tilde{D}_{\alpha\alpha}^{AA}(\boldsymbol{q}) - \tilde{D}_{\beta\beta}^{BB}(\boldsymbol{q})\right]^2 + \left[2\tilde{D}_{\alpha\beta}^{AB}(\boldsymbol{q})\right]^2}. \quad \text{(S9)}$$

For the B2 structure, there are 48 symmetry operations $\mathcal{S}$. By using the symmetry properties of the 180 degree rotations around x, y, and z axes and the inversion for the force constants [31], the expression of Eq. (S4) can be simplified. We introduce the step function $U(\xi)$ that takes 1 and 0 for $\xi \geq 0$ and $\xi < 0$, respectively. We obtain for $\alpha = x, y$, and z,

$$
\begin{aligned}
M_\kappa \tilde{D}^{\kappa\kappa}_{\alpha\alpha}(\boldsymbol{q}) = {} & D^{\kappa\kappa}_{\alpha\alpha}(\boldsymbol{0})U(p-1) \\
& + 2\left[-D^{\kappa\kappa}_{\alpha\alpha}(\boldsymbol{a}_1) - D^{\kappa\kappa}_{\alpha\alpha}(\boldsymbol{a}_2) + D^{\kappa\kappa}_{\alpha\alpha}(\boldsymbol{a}_3)\right]U(p-2) \\
& + 4\left[D^{\kappa\kappa}_{\alpha\alpha}(\boldsymbol{a}_1+\boldsymbol{a}_2) - D^{\kappa\kappa}_{\alpha\alpha}(\boldsymbol{a}_1+\boldsymbol{a}_3) - D^{\kappa\kappa}_{\alpha\alpha}(\boldsymbol{a}_2+\boldsymbol{a}_3)\right]U(p-3) \\
& + 8D^{\kappa\kappa}_{\alpha\alpha}(\boldsymbol{a}_1+\boldsymbol{a}_2+\boldsymbol{a}_3)U(p-5) \\
& + 2\left[D^{\kappa\kappa}_{\alpha\alpha}(2\boldsymbol{a}_1) + D^{\kappa\kappa}_{\alpha\alpha}(2\boldsymbol{a}_2) + D^{\kappa\kappa}_{\alpha\alpha}(2\boldsymbol{a}_3)\right]U(p-6)
\end{aligned} \tag{S10}
$$

The nonzero elements in the off-diagonal matrix for $(\alpha\beta) = (xy)$ and (yx) are

$$
\begin{aligned}
\sqrt{M_A M_B}\tilde{D}^{AB}_{\alpha\beta}(\boldsymbol{q}) = {} & 8D^{AB}_{\alpha\beta}(\boldsymbol{0})U(p-1) \\
& + 8\left[-D^{AB}_{\alpha\beta}(2\boldsymbol{a}_1+\boldsymbol{a}_2+\boldsymbol{a}_3) - D^{AB}_{\alpha\beta}(\boldsymbol{a}_1+2\boldsymbol{a}_2+\boldsymbol{a}_3) + D^{AB}_{\alpha\beta}(\boldsymbol{a}_1+\boldsymbol{a}_2+2\boldsymbol{a}_3)\right]U(p-4)
\end{aligned} \tag{S11}
$$

and

$$
\begin{aligned}
\sqrt{M_A M_B}\tilde{D}^{BA}_{\alpha\beta}(\boldsymbol{q}) = {} & 8D^{BA}_{\alpha\beta}(\boldsymbol{0})U(p-1) \\
& + 8\left[-D^{BA}_{\alpha\beta}(\boldsymbol{a}_1) - D^{BA}_{\alpha\beta}(\boldsymbol{a}_2) + D^{BA}_{\alpha\beta}(\boldsymbol{a}_3)\right]U(p-4).
\end{aligned} \tag{S12}
$$

The acoustic sum rule determines $D^{\kappa\kappa}_{\alpha\alpha}(\boldsymbol{0})$ in Eqs. (S10), which is expressed by

$$
\begin{aligned}
D^{AA}_{\alpha\alpha}(\boldsymbol{0}) = {} & -8D^{BA}_{\alpha\alpha}(\boldsymbol{0})U(p-1) \\
& - 2\left[D^{AA}_{\alpha\alpha}(\boldsymbol{a}_1) + D^{AA}_{\alpha\alpha}(\boldsymbol{a}_2) + D^{AA}_{\alpha\alpha}(\boldsymbol{a}_3)\right]U(p-2) \\
& - 4\left[D^{AA}_{\alpha\alpha}(\boldsymbol{a}_1+\boldsymbol{a}_2) + D^{AA}_{\alpha\alpha}(\boldsymbol{a}_1+\boldsymbol{a}_3) + D^{AA}_{\alpha\alpha}(\boldsymbol{a}_2+\boldsymbol{a}_3)\right]U(p-3) \\
& - 8\left[D^{BA}_{\alpha\beta}(\boldsymbol{a}_1) + D^{BA}_{\alpha\beta}(\boldsymbol{a}_2) + D^{BA}_{\alpha\beta}(\boldsymbol{a}_3)\right]U(p-4) \\
& - 8D^{AA}_{\alpha\alpha}(\boldsymbol{a}_1+\boldsymbol{a}_2+\boldsymbol{a}_3)U(p-5) \\
& - 2\left[D^{AA}_{\alpha\alpha}(2\boldsymbol{a}_1) + D^{AA}_{\alpha\alpha}(2\boldsymbol{a}_2) + D^{AA}_{\alpha\alpha}(2\boldsymbol{a}_3)\right]U(p-6)
\end{aligned} \tag{S13}
$$

and

$$
\begin{aligned}
D^{BB}_{\alpha\alpha}(\boldsymbol{0}) = {} & -8D^{AB}_{\alpha\alpha}(\boldsymbol{0})U(p-1) \\
& - 2\left[D^{BB}_{\alpha\alpha}(\boldsymbol{a}_1) + D^{BB}_{\alpha\alpha}(\boldsymbol{a}_2) + D^{BB}_{\alpha\alpha}(\boldsymbol{a}_3)\right]U(p-2) \\
& - 4\left[D^{BB}_{\alpha\alpha}(\boldsymbol{a}_1+\boldsymbol{a}_2) + D^{BB}_{\alpha\alpha}(\boldsymbol{a}_1+\boldsymbol{a}_3) + D^{BB}_{\alpha\alpha}(\boldsymbol{a}_2+\boldsymbol{a}_3)\right]U(p-3) \\
& - 8\left[D^{AB}_{\alpha\beta}(2\boldsymbol{a}_1+\boldsymbol{a}_2+\boldsymbol{a}_3) + D^{AB}_{\alpha\beta}(\boldsymbol{a}_1+2\boldsymbol{a}_2+\boldsymbol{a}_3) + D^{AB}_{\alpha\beta}(\boldsymbol{a}_1+\boldsymbol{a}_2+2\boldsymbol{a}_3)\right]U(p-4) \\
& - 8D^{BB}_{\alpha\alpha}(\boldsymbol{a}_1+\boldsymbol{a}_2+\boldsymbol{a}_3)U(p-5) \\
& - 2\left[D^{BB}_{\alpha\alpha}(2\boldsymbol{a}_1) + D^{BB}_{\alpha\alpha}(2\boldsymbol{a}_2) + D^{BB}_{\alpha\alpha}(2\boldsymbol{a}_3)\right]U(p-6).
\end{aligned} \tag{S14}
$$

TABLE S1. The 416 B2 compounds *AB*. The dynamically stable compounds are shown in bold.

A	*AB* or *BA*
H	**CsH**, NaH
Li	**LiAg, LiBe**, LiF, **LiHg, LiPb, LiPd, LiTl**
Na	NaCl, NaH, NaS
K	**KBr, KCl, KF, KI**, KS
Rb	**RbAu, RbBr, RbCl**, RbF, **RbI**, RbS
Cs	**CsAu, CsBr, CsCl**, CsF, **CsH, CsI**, CsN, CsS, CsSe
Be	**BeCo, BeCu, BeNi, BePd, BeRh, LiBe, TiBe**
Mg	**CeMg, DyMg, ErMg**, EuMg, **GdMg, HoMg**, LaMg, LuMg, **MgAg, MgAu**, MgHg, **MgNi**, MgO, **MgPd, MgRh**, **MgSc**, MgTl, **NdMg, PmMg**, PrMg, **SmMg**, SrMg, **TbMg, TmMg**, YMg
Ca	**CaCd, CaHg, CaIn, CaNi, CaPd**, CaTe, CaTl
Sr	**SrCd, SrHg**, SrMg, SrO, SrS, SrTe, **SrTl**
Ba	**BaCd, BaHg**, BaSe, **BaTe, BaZn**
Sc	**MgSc, ScAg, ScAl**, ScAs, **ScAu**, ScC, **ScCd, ScCo, ScCu, ScHg, ScIr**, ScN, **ScNi**, ScP, **ScPd, ScPt, ScRh**, **ScRu**, ScSb, **ScZn**
Y	**YAg, YAl**, YAs, **YAu**, YC, **YCd, YCu, YHg, YIn, YIr**, YMg, YN, YP, **YRh**, YSb, YTi, **YTl, YZn**
Ti	TiAl, TiAu, **TiBe, TiCo, TiFe**, TiIr, TiN, TiNi, **TiOs**, TiPd, TiPt, **TiRe**, TiRh, **TiRu, TiTc**, YTi
Zr	ZrC, **ZrCo**, ZrCu, ZrIr, ZrN, **ZrOs**, ZrPd, ZrPt, ZrRh, **ZrRu, ZrZn**
Hf	HfC, **HfCo**, HfIr, HfN, **HfOs**, HfPd, HfPt, **HfRh, HfRu, HfTc**
V	**MnV, VCo, VFe**, VN, **VOs**, VRu, **VTc**
Nb	NbC, **NbCo, NbN**, NbRu
Ta	TaC, **TaCo, TaN**, TaRu, **TaTc**
Cr	**CrCo**, CrN
Mo	CoMo, MoN
W	CoW, WC
Mn	MnAu, **MnCo**, MnHg, MnN, **MnNi, MnPd, MnRh**, MnS, MnSb, MnSn, **MnV, MnZn**
Tc	**HfTc, TaTc**, TcB, TcN, **TiTc, VTc**
Re	AlRe, ReC, ReN, **TiRe**
Fe	**AlFe, FeCo**, FeN, **FeRh**, FeSe, **TiFe, VFe**
Ru	**AlRu, GaRu, HfRu, LuRu**, NbRu, **ScRu, SiRu**, TaRu, **TiRu**, VRu, **YbRu, ZrRu**
Os	**AlOs, HfOs**, OsC, **SiOs, TiOs, VOs, ZrOs**
Co	**AlCo, BeCo**, CoMo, CoN, CoNi, **CoSi**, CoW, **CrCo, FeCo, GaCo, HfCo, MnCo, NbCo, ScCo, TaCo, TiCo**, **VCo, ZrCo**
Rh	**AlRh, BeRh, DyRh, ErRh, FeRh, GaRh, GdRh, HfRh, HoRh, InRh, LuRh, MgRh, MnRh, ScRh**, SiRh, **SmRh, TbRh**, TiRh, **TmRh, YRh, YbRh, ZnRh**, ZrRh
Ir	**AlIr, ErIr, GaIr**, HfIr, **HoIr**, IrC, IrN, **LuIr, ScIr**, TiIr, **TmIr, YIr, YbIr**, ZrIr
Ni	**AlNi, BeNi, CaNi**, CoNi, **GaNi, InNi, MgNi, MnNi**, NiN, **ScNi**, TiNi, **ZnNi**
Pd	**AlPd, BePd, CaPd, CuPd, DyPd, ErPd**, HfPd, **HoPd, InPd, LiPd, LuPd, MgPd, MnPd**, PdN, PdO, **ScPd**, TiPd, **TmPd, YbPd**, ZrPd
Pt	AlPt, HfPt, PtC, **ScPt, TbPt**, TiPt, ZrPt
Cu	**BeCu**, CuN, **CuPd, DyCu, ErCu, EuCu, GdCu, HoCu, ScCu, SmCu, TbCu, TmCu, YCu**, ZnCu, ZrCu
Ag	AgI, CdAg, **CeAg, DyAg, ErAg**, GaAg, **GdAg, HoAg**, LaAg, **LiAg, MgAg, NdAg, PrAg, ScAg, SmAg**, **TbAg, TmAg, YAg, YbAg, ZnAg**
Au	AlAu, CdAu, **CsAu, DyAu, ErAu, GdAu, HoAu, LuAu, MgAu**, MnAu, **NdAu, PrAu, RbAu, ScAu, SmAu**, **TbAu**, TiAu, **TmAu, YAu, YbAu, ZnAu**
Zn	**BaZn**, CeZn, **DyZn, ErZn, EuZn, GdZn, HoZn**, LaZn, **LuZn, MnZn**, NdZn, PrZn, **ScZn, SmZn, TbZn**, **TmZn, YZn, YbZn, ZnAg, ZnAu**, ZnCu, ZnN, **ZnNi**, ZnO, **ZnRh, ZrZn**
Cd	**BaCd, CaCd**, CdAg, CdAu, CdN, **CeCd, DyCd, ErCd, EuCd, GdCd, HoCd, LaCd, LuCd, NdCd, PrCd**, **ScCd, SmCd, SrCd, TbCd, TmCd, YCd, YbCd**
Hg	**BaHg, CaHg, CeHg, DyHg, ErHg, EuHg, GdHg**, HgTe, **HoHg, LaHg, LiHg, LuHg**, MgHg, MnHg, **NdHg**, **PrHg, ScHg, SrHg, TbHg, TlHg, TmHg, YHg, YbHg**

TABLE S1. *Continued.*

A	*AB* or *BA*
B	TcB
Al	AlAu, **AlCo, AlFe, AlIr, AlNi, AlOs, AlPd**, AlPt, AlRe, **AlRh, AlRu, CeAl, DyAl, GdAl, NdAl, PrAl**, **ScAl, SmAl, TbAl**, TiAl, **YAl**
Ga	GaAg, **GaCo, GaIr, GaNi, GaRh, GaRu**
In	**CaIn, DyIn, ErIn, GdIn, HoIn, InNi, InPd, InRh**, InSb, InTe, **LaIn, PrIn, SmIn, TmIn, YIn, YbIn**
Tl	**CaTl, DyTl, ErTl, EuTl, GdTl, HoTl, LaTl, LiTl**, MgTl, **NdTl, PrTl, SmTl, SrTl, TbTl**, TlBi, **TlBr**, TlC, TlCl, **TlHg**, TlI, TlS, TlSb, TlSe, **TmTl, YTl, YbTl**
C	HfC, IrC, NbC, OsC, PtC, ReC, ScC, TaC, TlC, WC, YC, ZrC
Si	**CoSi, SiOs**, SiRh, **SiRu**
Ge	GeTe
Sn	MnSn, SnSb, SnTe
Pb	**LiPb**, PbS, PbSe, TePb
N	BrN, CdN, CoN, CrN, CsN, CuN, ErN, FeN, HfN, HoN, IN, IrN, LaN, MnN, MoN, NCl, **NbN**, NdN, NiN, PdN, PrN, ReN, ScN, **TaN**, TcN, TiN, VN, YN, ZnN, ZrN
P	CeP, DyP, ErP, HoP, PBr, ScP, YP
As	DyAs, ErAs, HoAs, LuAs, ScAs, YAs
Sb	DySb, ErSb, HoSb, InSb, LuSb, MnSb, ScSb, SnSb, TlSb, TmSb, YSb
Bi	CeBi, HoBi, PrBi, TlBi
O	ClO, MgO, PdO, SrO, ZnO
S	**CeS**, CsS, EuS, KS, **LaS**, MnS, NaS, PbS, RbS, SrS, TlS
Se	BaSe, **CeSe**, CsSe, **EuSe**, FeSe, **LaSe**, PbSe, TlSe
Te	**BaTe**, CaTe, **CeTe**, EuTe, GeTe, HgTe, InTe, **LaTe, PrTe, SmTe**, SnTe, SrTe, TePb, **TmTe**
F	CsF, **KF**, LiF, RbF
Cl	ClO, **CsCl, KCl**, NCl, NaCl, **RbCl**, TlCl
Br	BrN, **CsBr, KBr**, PBr, **RbBr, TlBr**
I	AgI, **CsI**, IN, **KI, RbI**, TlI
La	LaAg, **LaCd, LaHg, LaIn**, LaMg, LaN, **LaS, LaSe, LaTe, LaTl**, LaZn
Ce	**CeAg, CeAl**, CeBi, **CeCd, CeHg, CeMg**, CeP, **CeS, CeSe, CeTe**, CeZn
Pr	**PrAg, PrAl, PrAu**, PrBi, **PrCd, PrHg, PrIn**, PrMg, PrN, **PrTe, PrTl**, PrZn
Nd	**NdAg, NdAl, NdAu, NdCd, NdHg, NdMg**, NdN, **NdTl**, NdZn
Pm	**PmMg**
Sm	**SmAg, SmAl, SmAu, SmCd, SmCu, SmIn, SmMg, SmRh, SmTe, SmTl, SmZn**
Eu	**EuCd, EuCu, EuHg**, EuMg, EuS, **EuSe**, EuTe, **EuTl, EuZn**
Gd	**GdAg, GdAl, GdAu, GdCd, GdCu, GdHg, GdIn, GdMg, GdRh, GdTl, GdZn**
Tb	**TbAg, TbAl, TbAu, TbCd, TbCu, TbHg, TbMg, TbPt, TbRh, TbTl, TbZn**
Dy	**DyAg, DyAl**, DyAs, **DyAu, DyCd, DyCu, DyHg, DyIn, DyMg**, DyP, **DyPd, DyRh**, DySb, **DyTl, DyZn**
Ho	**HoAg**, HoAs, **HoAu**, HoBi, **HoCd, HoCu, HoHg, HoIn, HoIr, HoMg**, HoN, HoP, **HoPd, HoRh**, HoSb, **HoTl, HoZn**
Er	**ErAg**, ErAs, **ErAu, ErCd, ErCu, ErHg, ErIn, ErIr, ErMg**, ErN, ErP, **ErPd, ErRh**, ErSb, **ErTl, ErZn**
Tm	**TmAg, TmAu, TmCd, TmCu, TmHg, TmIn, TmIr, TmMg, TmPd, TmRh**, TmSb, **TmTe, TmTl, TmZn**
Yb	**YbAg, YbAu, YbCd, YbHg, YbIn, YbIr, YbPd, YbRh, YbRu, YbTl, YbZn**
Lu	LuAs, **LuAu, LuCd, LuHg, LuIr**, LuMg, **LuPd, LuRh, LuRu**, LuSb, **LuZn**

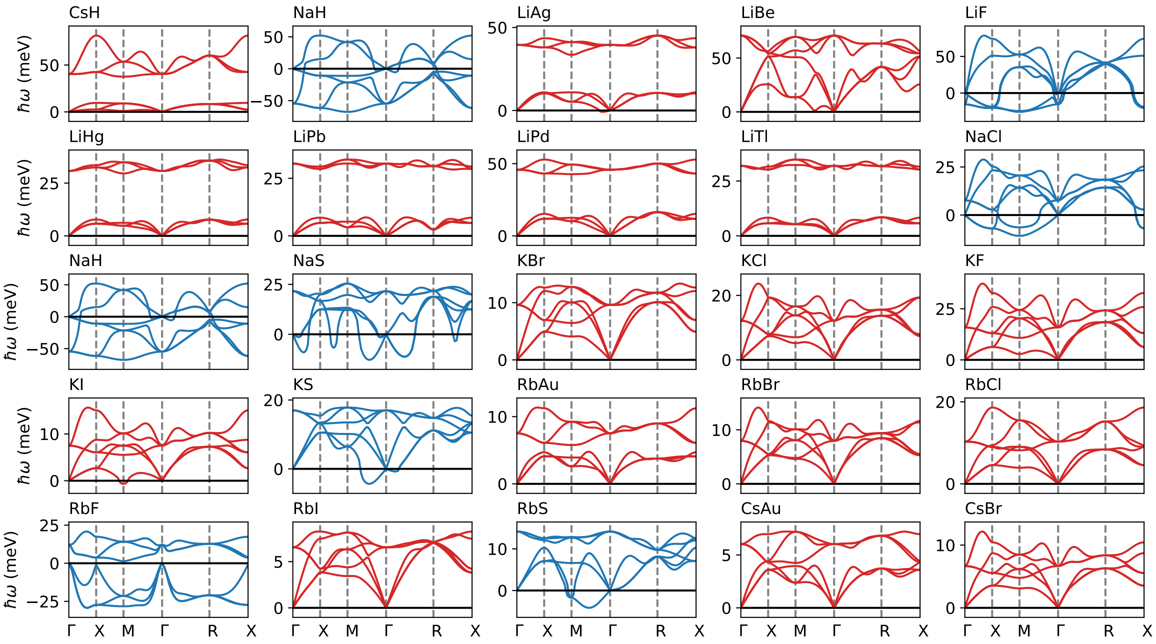

FIG. S1. The phonon dispersions for 416 B2 compounds. The curves are colored red and blue for the stable and unstable compounds, respectively.

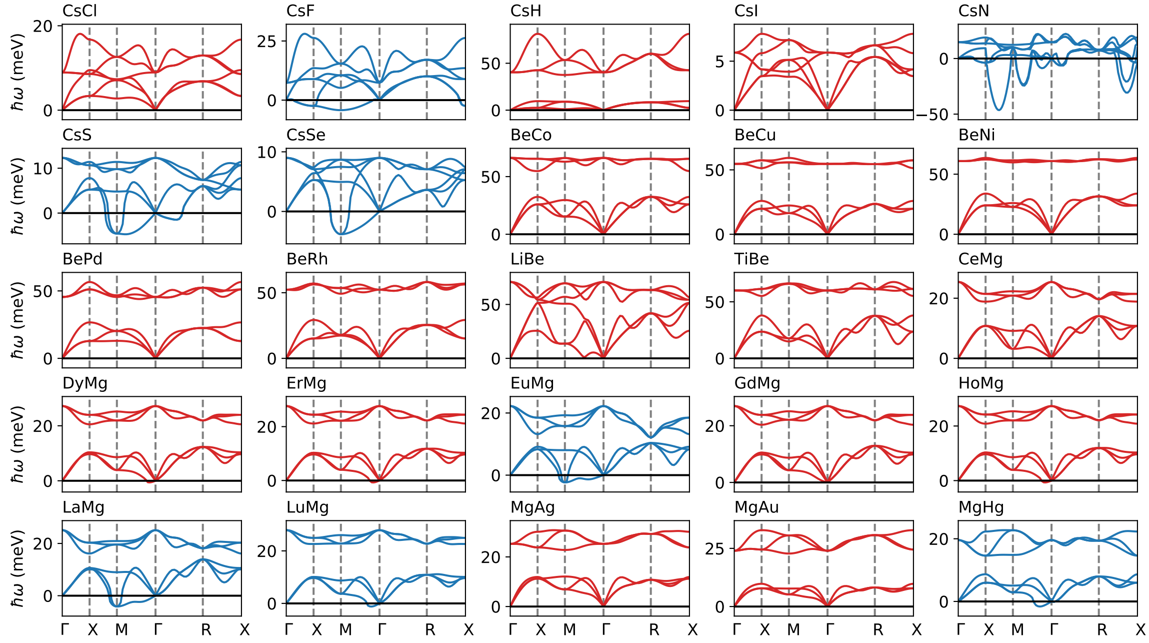

FIG. S1. *Continued.*

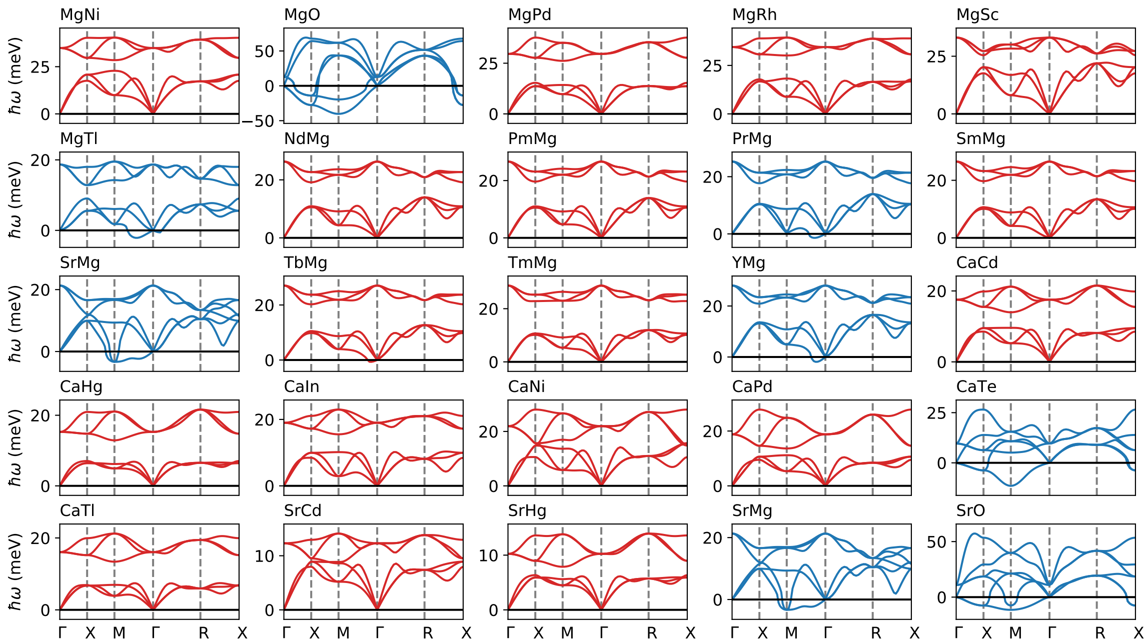

FIG. S1. *Continued.*

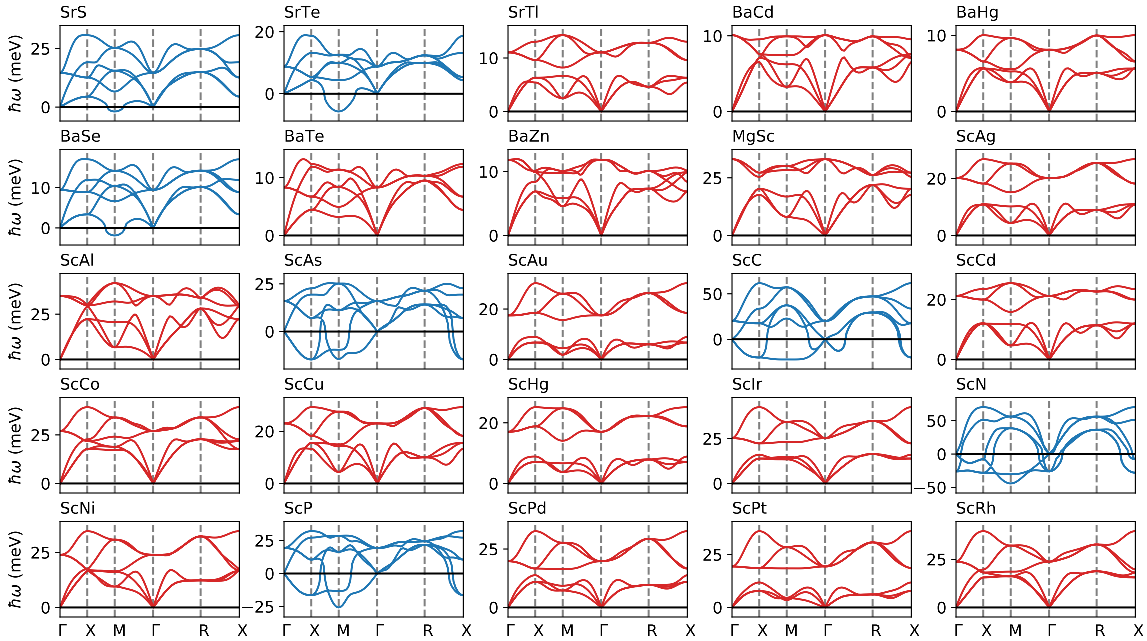

FIG. S1. *Continued.*

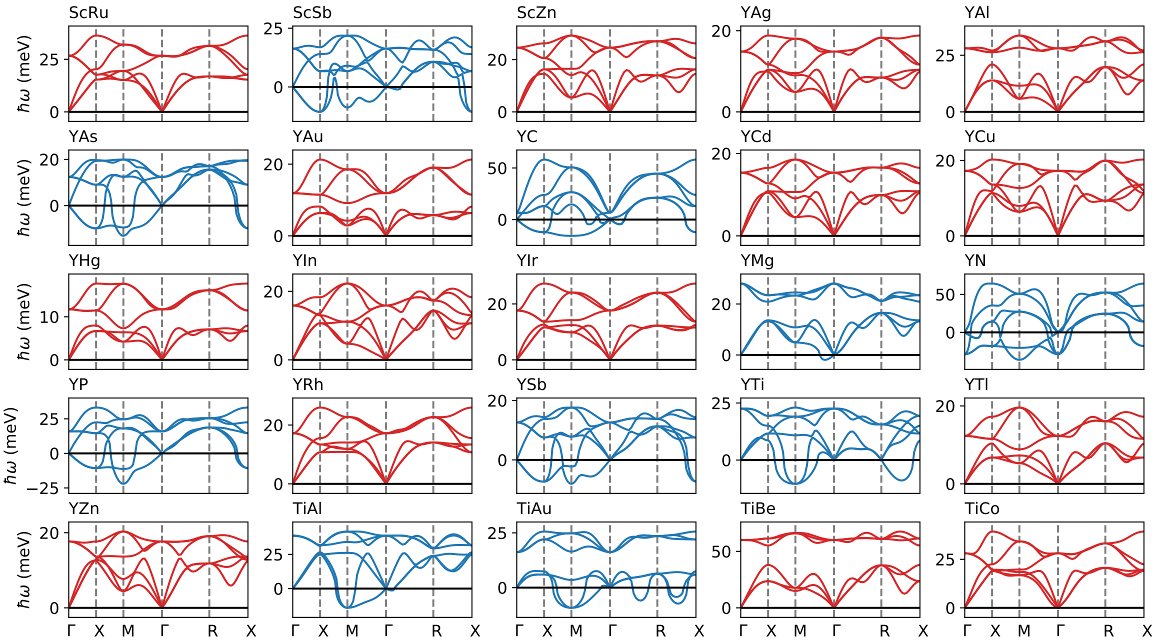

FIG. S1. *Continued.*

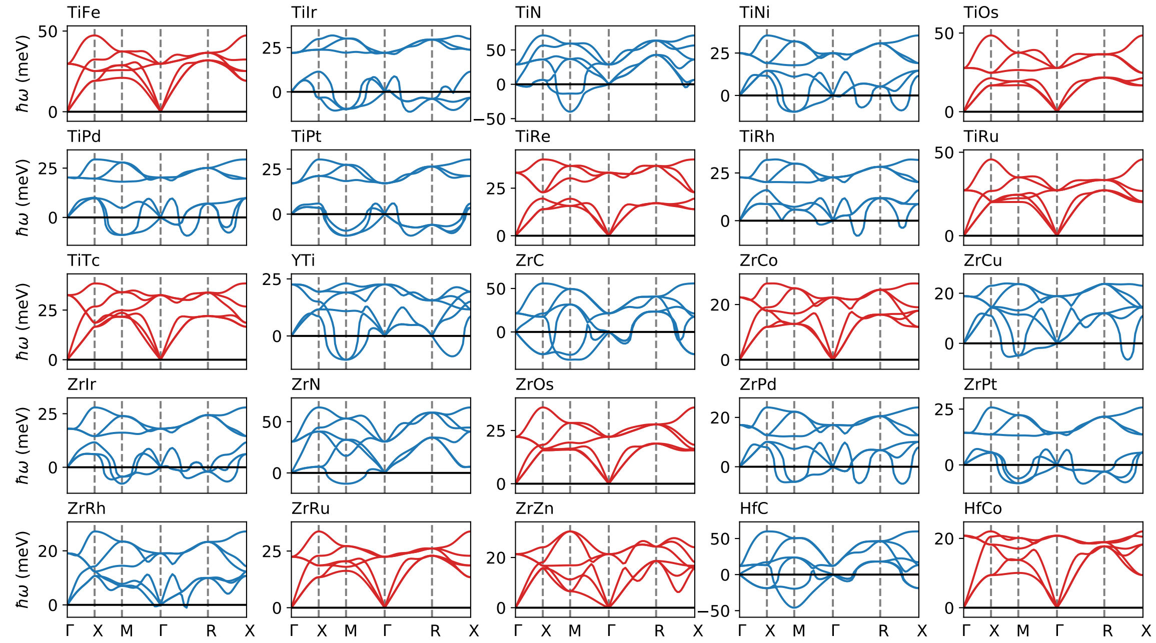

FIG. S1. *Continued.*

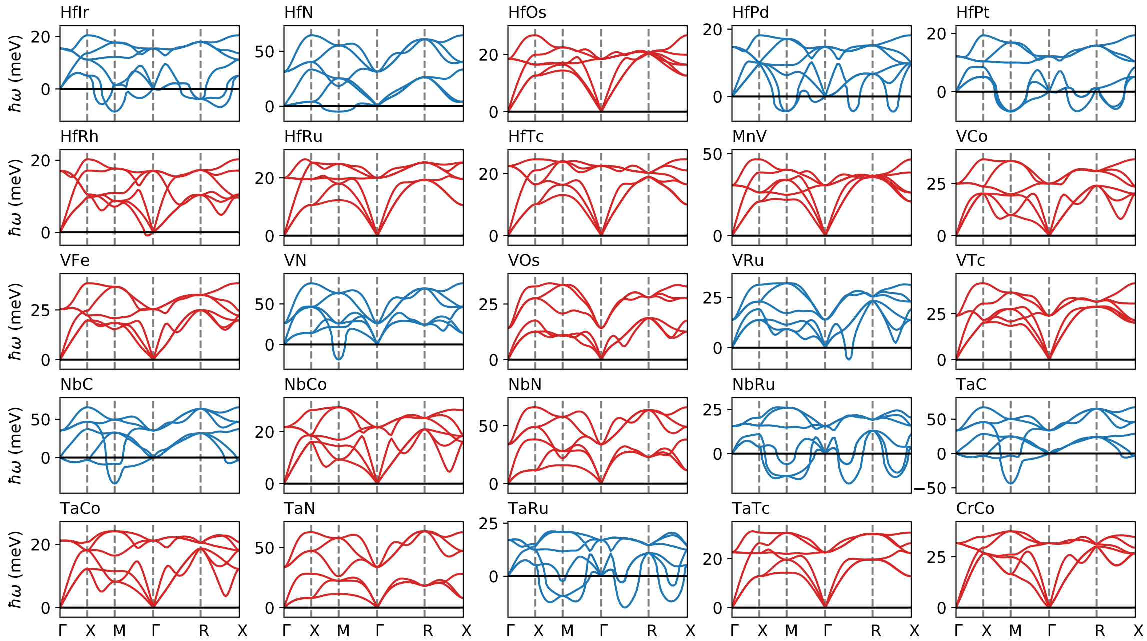

FIG. S1. *Continued.*

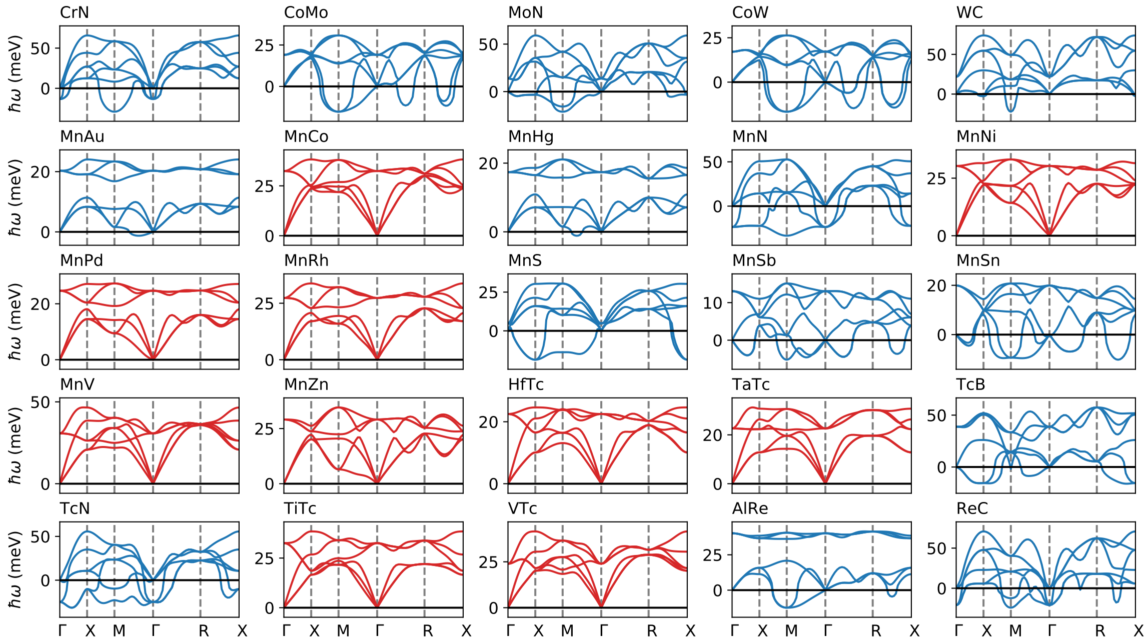

FIG. S1. *Continued.*

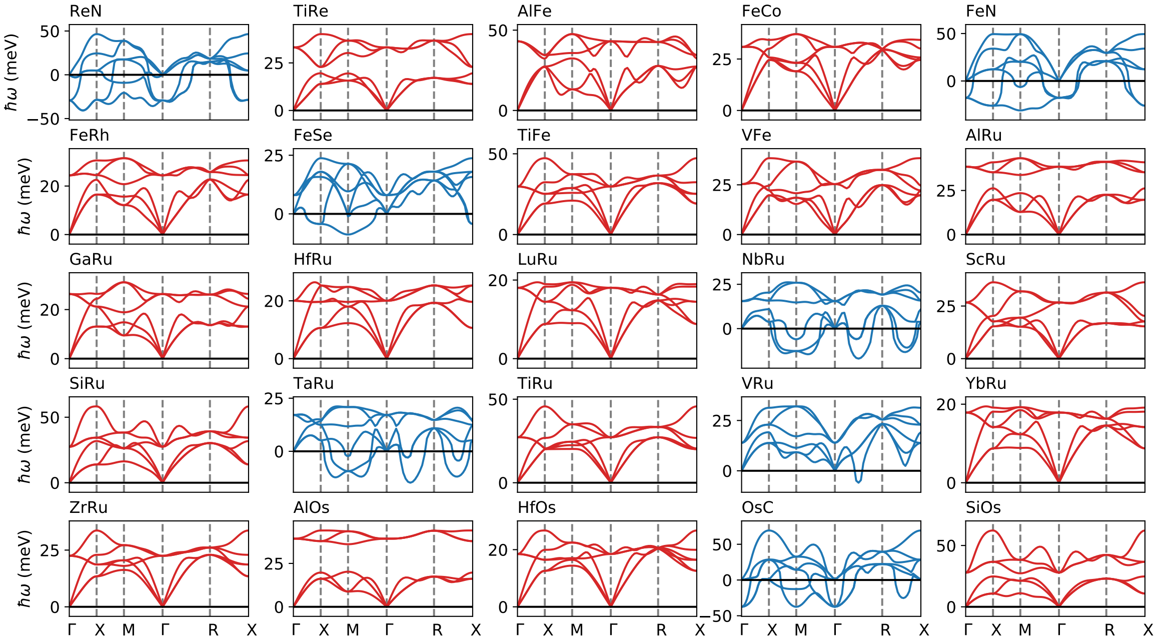

FIG. S1. *Continued.*

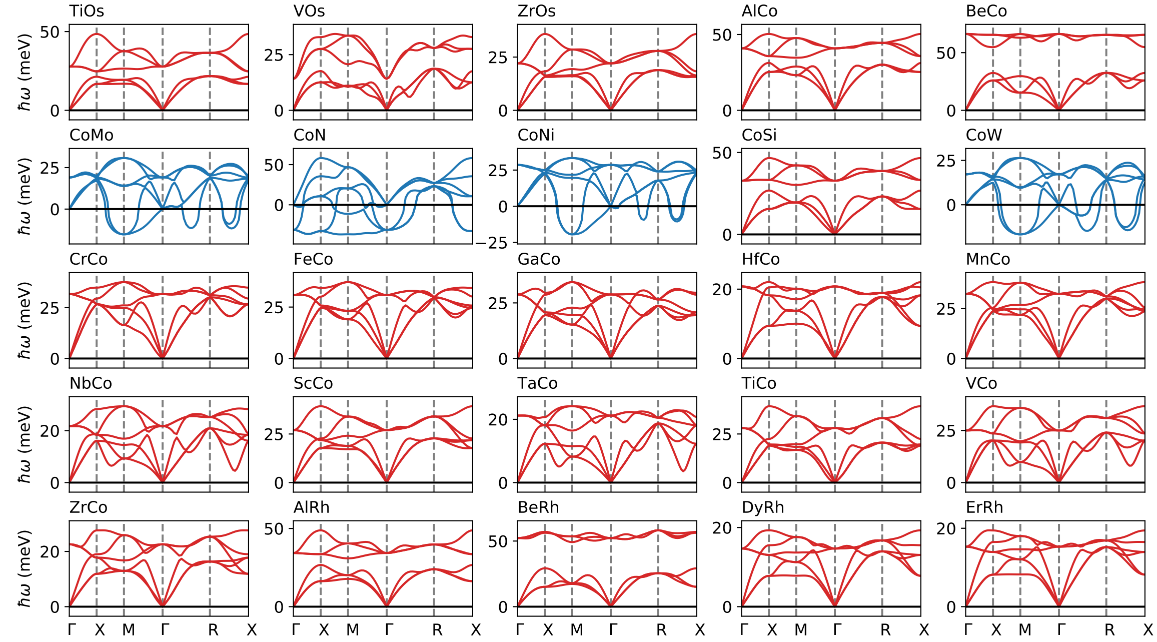

FIG. S1. *Continued.*

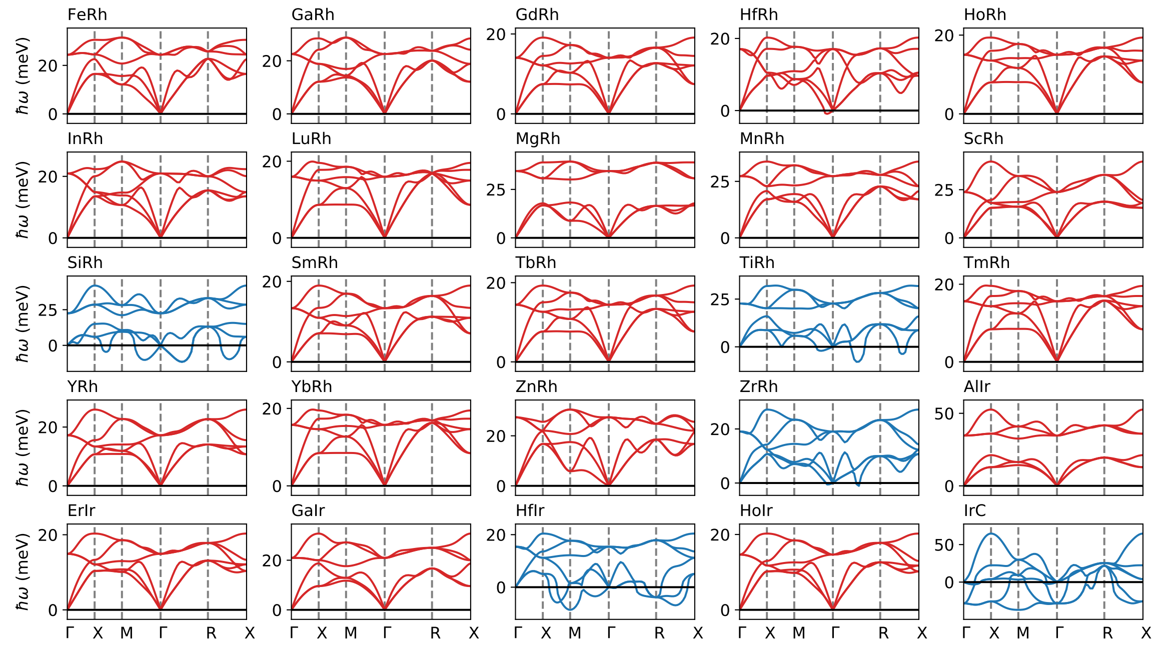

FIG. S1. *Continued.*

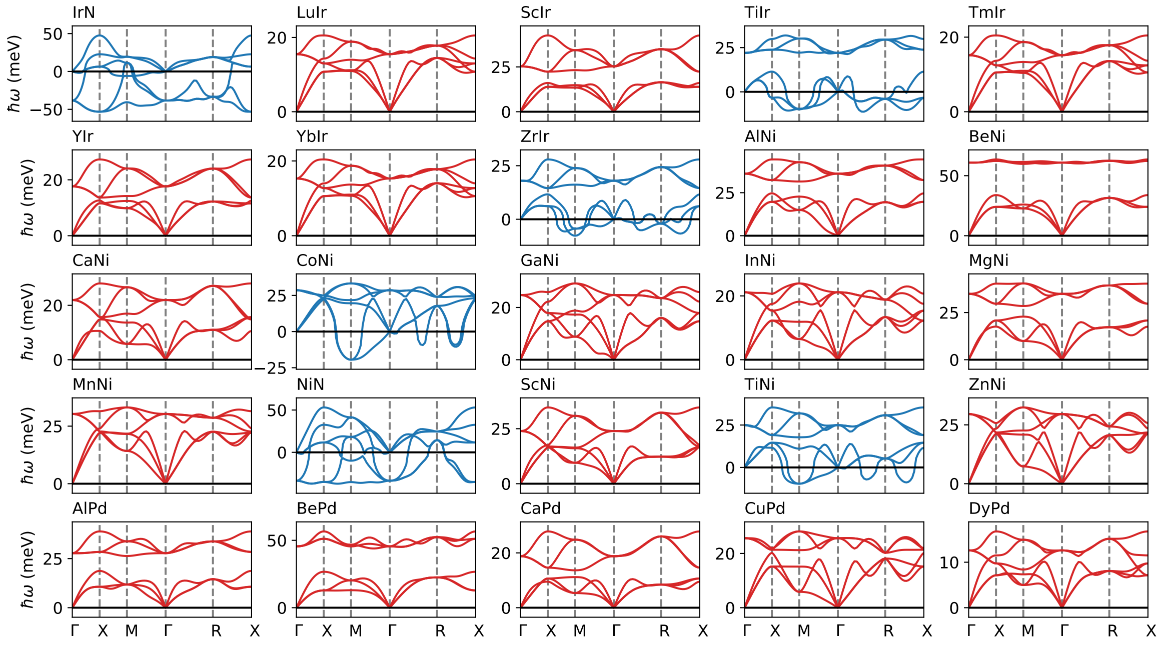

FIG. S1. *Continued.*

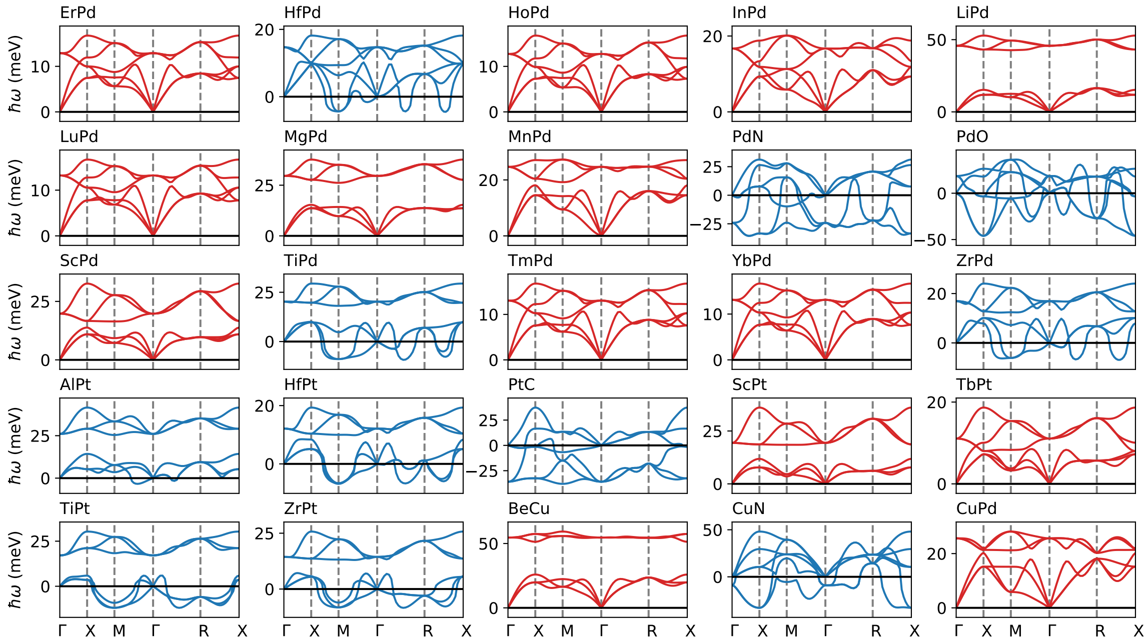

FIG. S1. *Continued.*

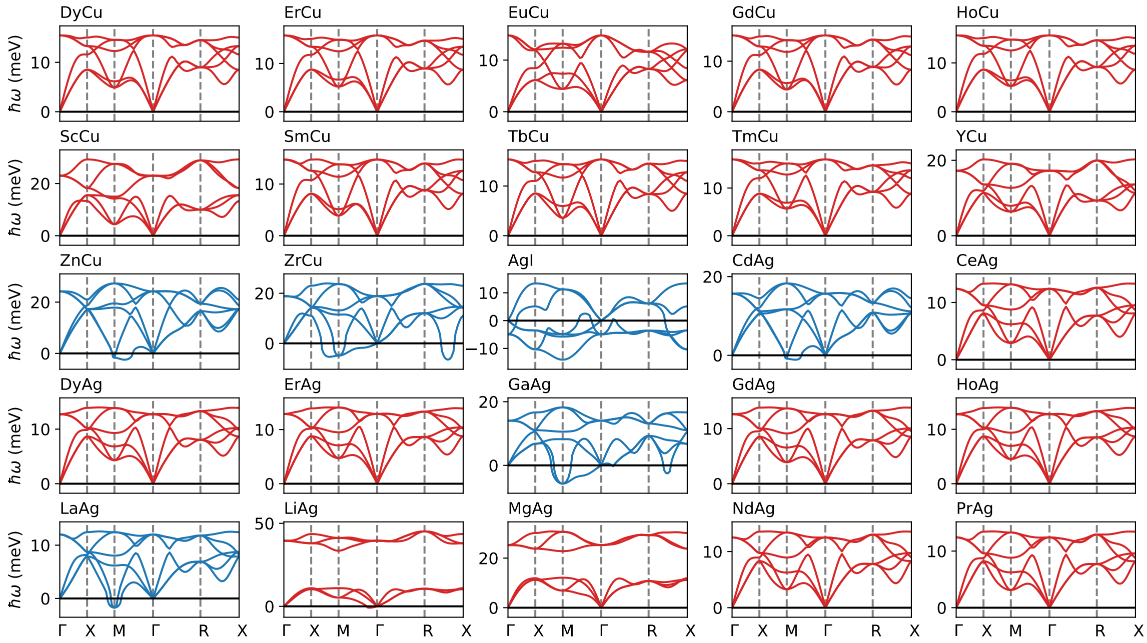

FIG. S1. *Continued.*

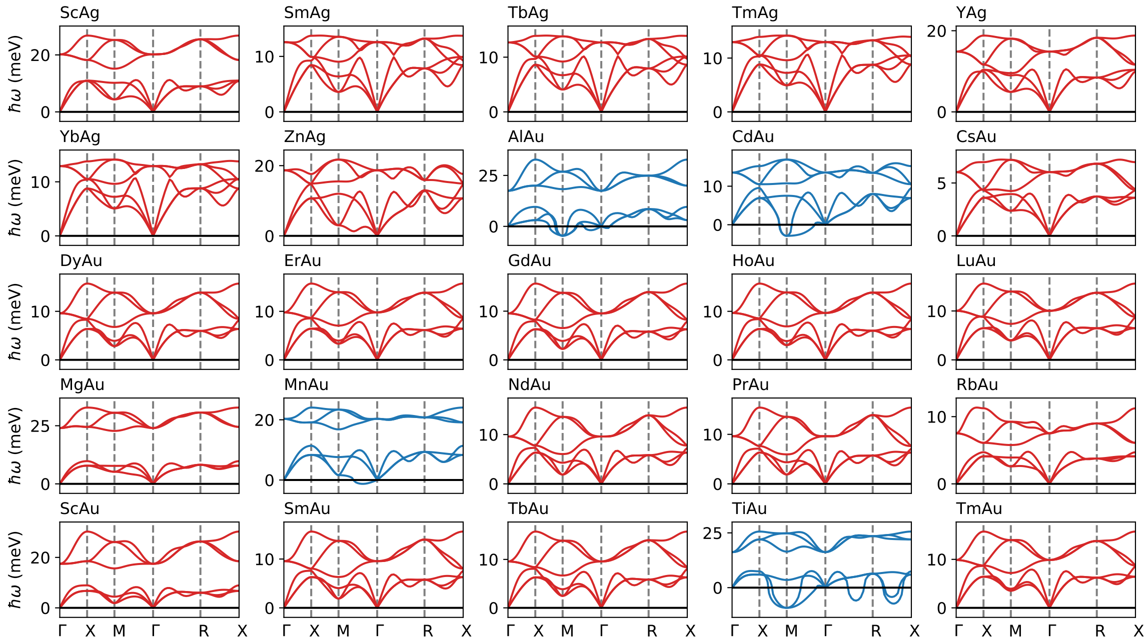

FIG. S1. *Continued.*

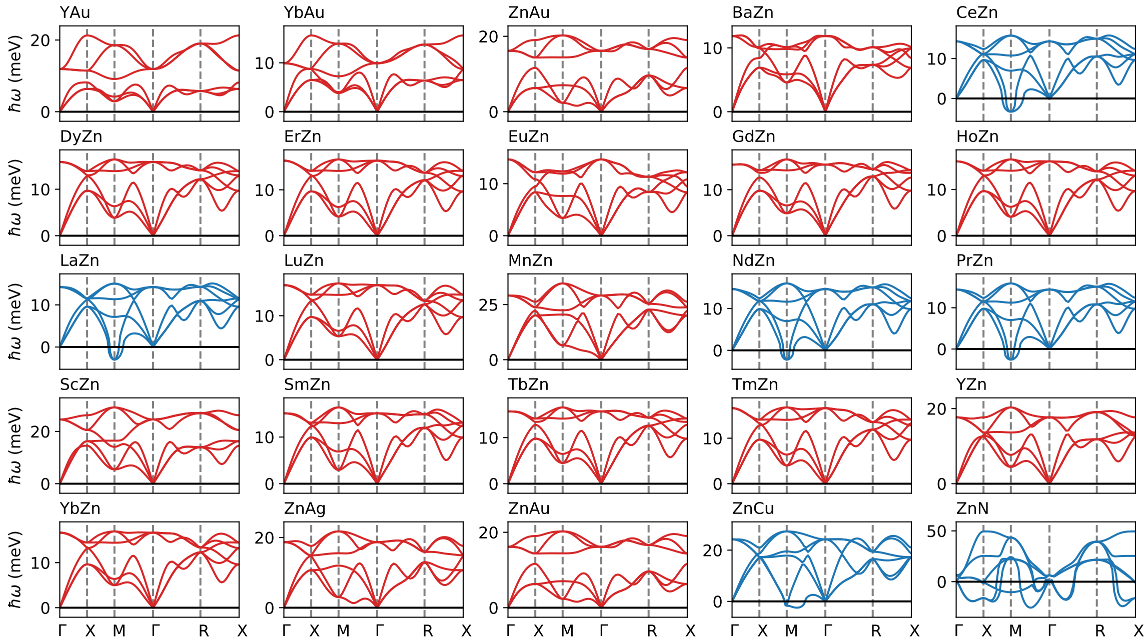

FIG. S1. *Continued.*

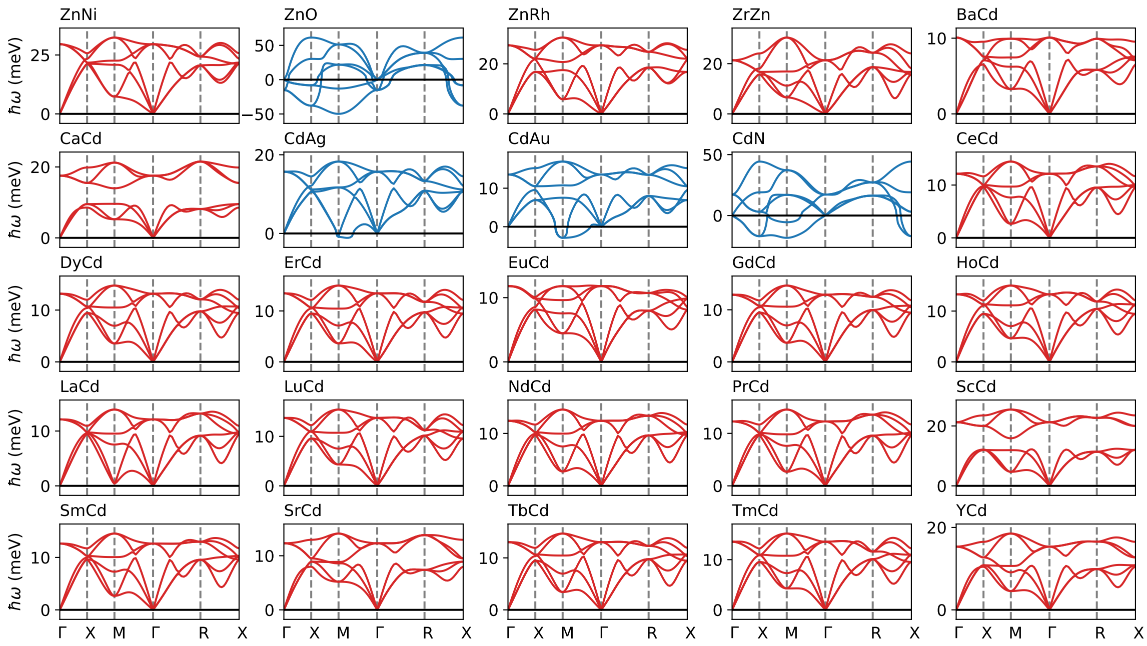

FIG. S1. *Continued.*

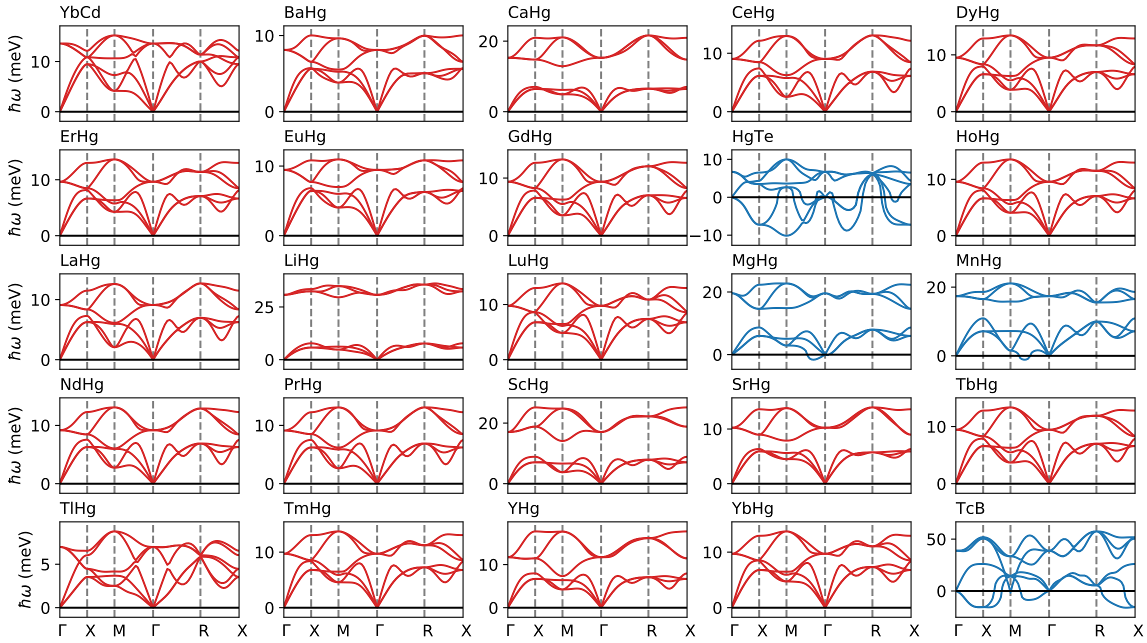

FIG. S1. *Continued.*

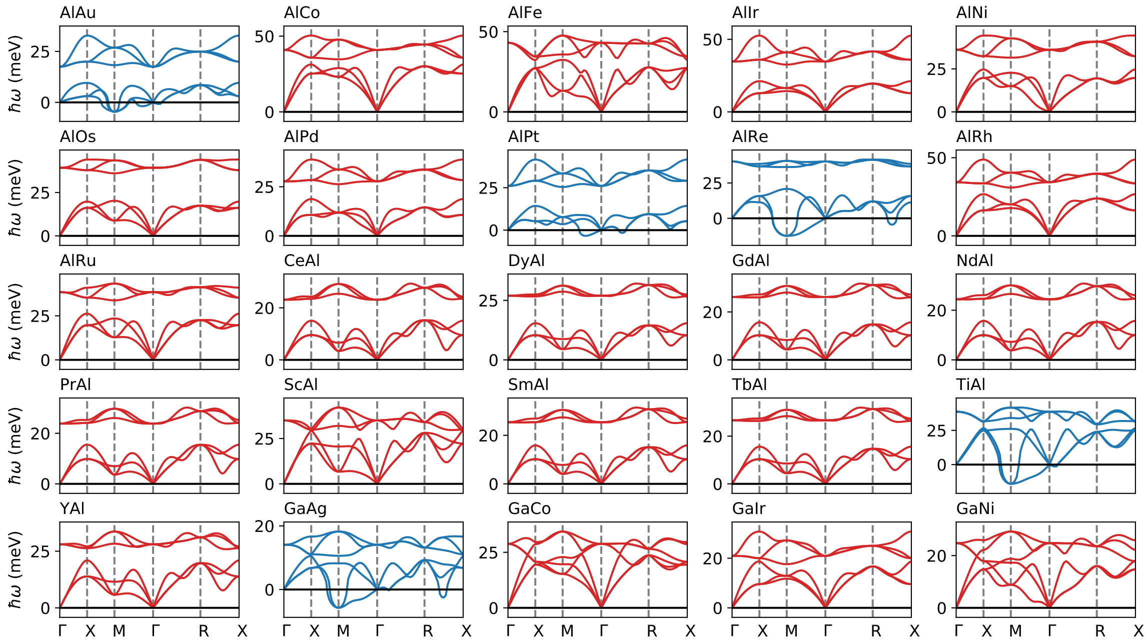

FIG. S1. *Continued.*

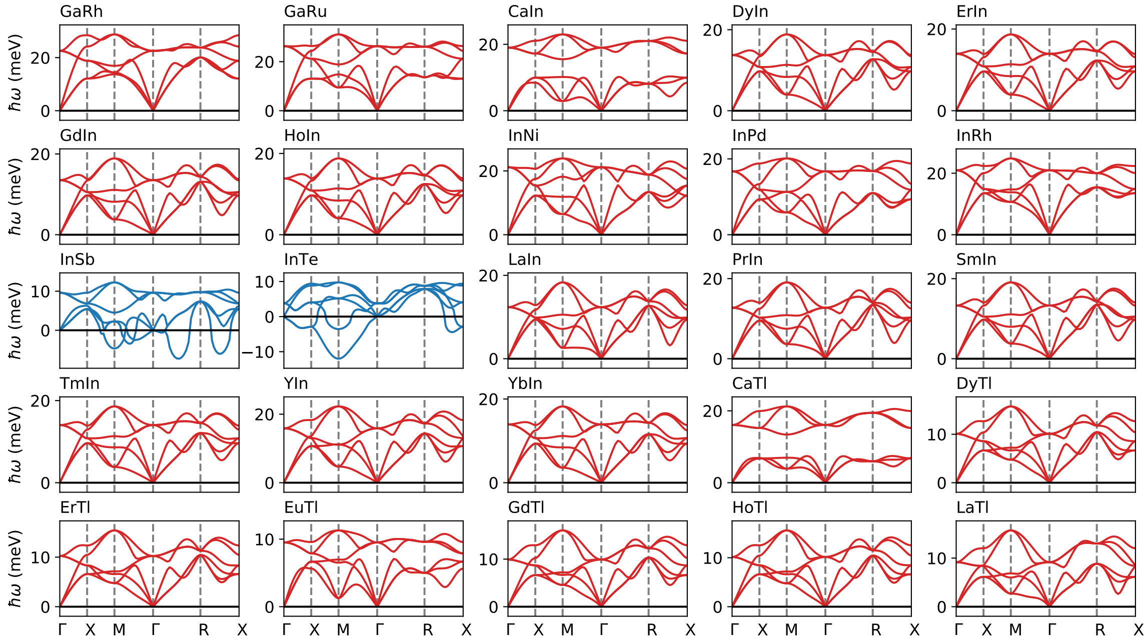

FIG. S1. *Continued.*

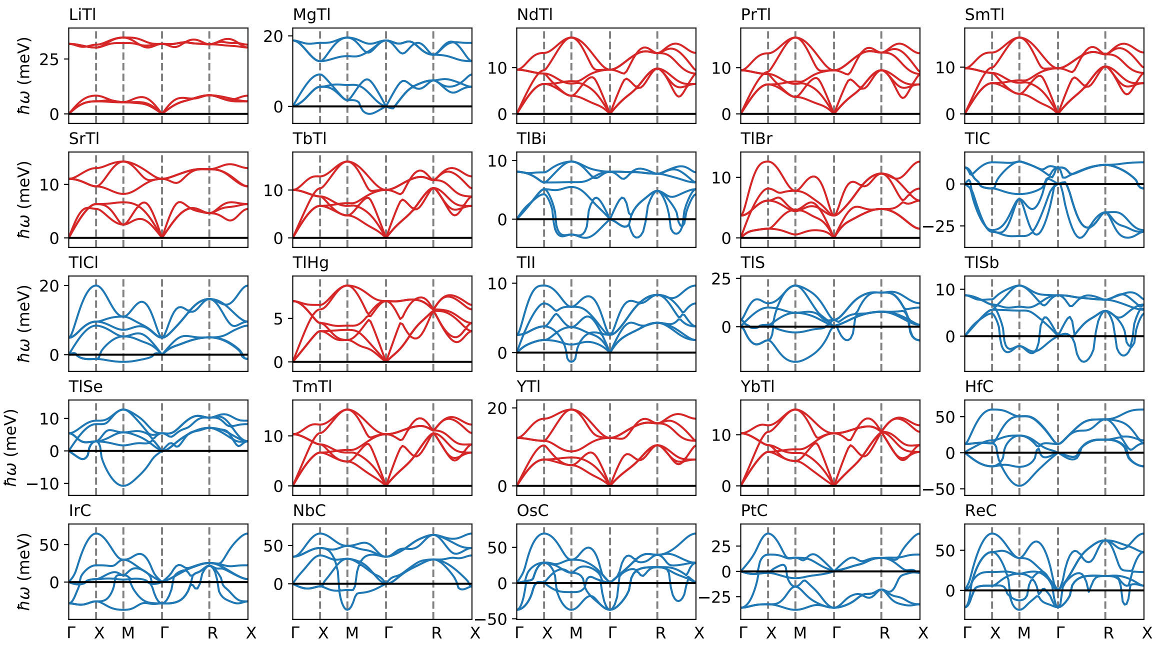

FIG. S1. *Continued.*

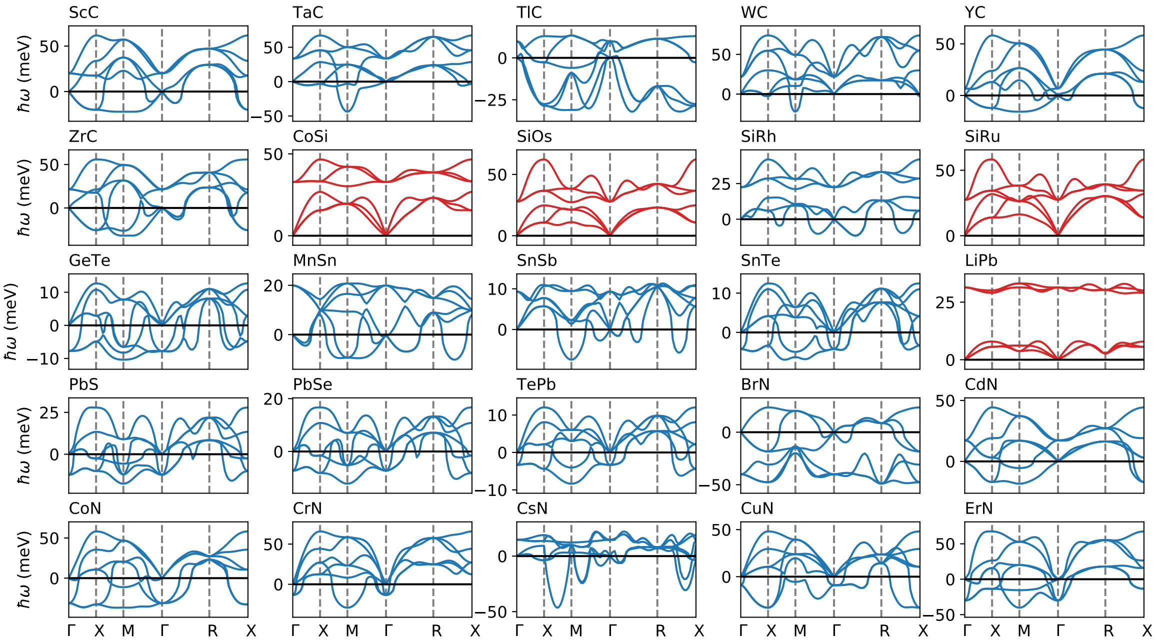

FIG. S1. *Continued.*

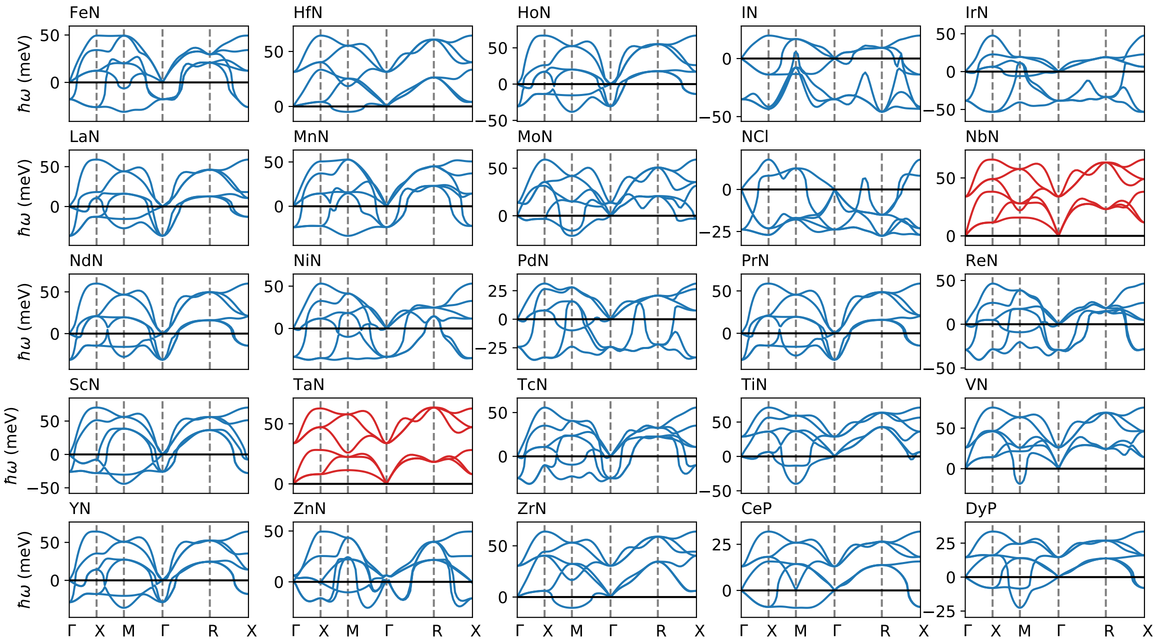

FIG. S1. *Continued.*

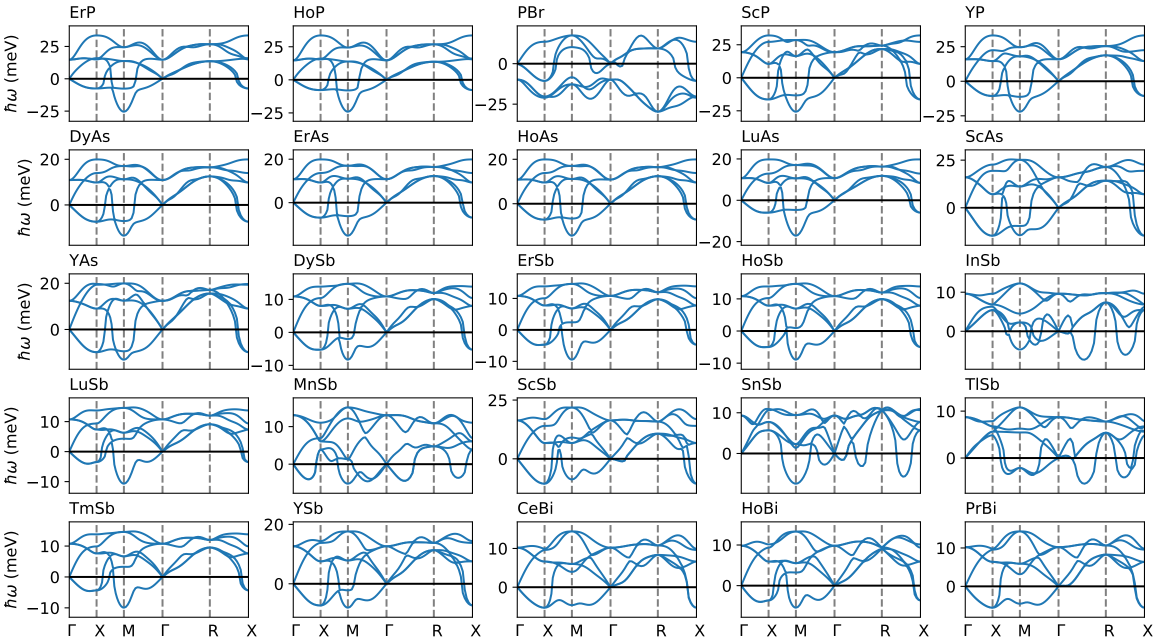

FIG. S1. *Continued.*

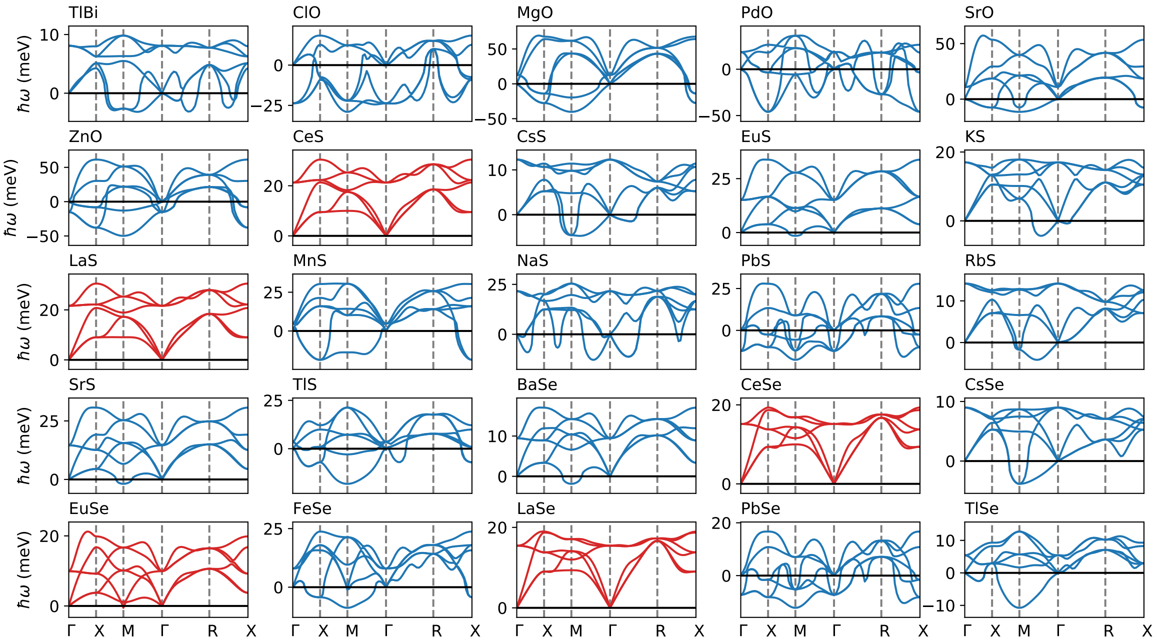

FIG. S1. *Continued.*

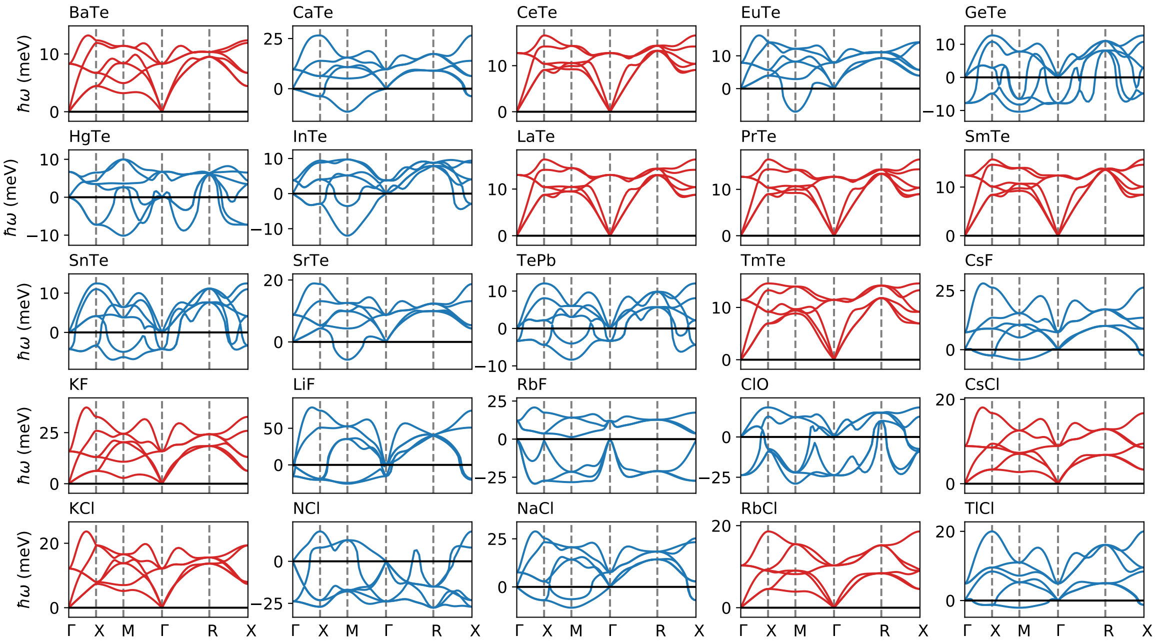

FIG. S1. *Continued.*

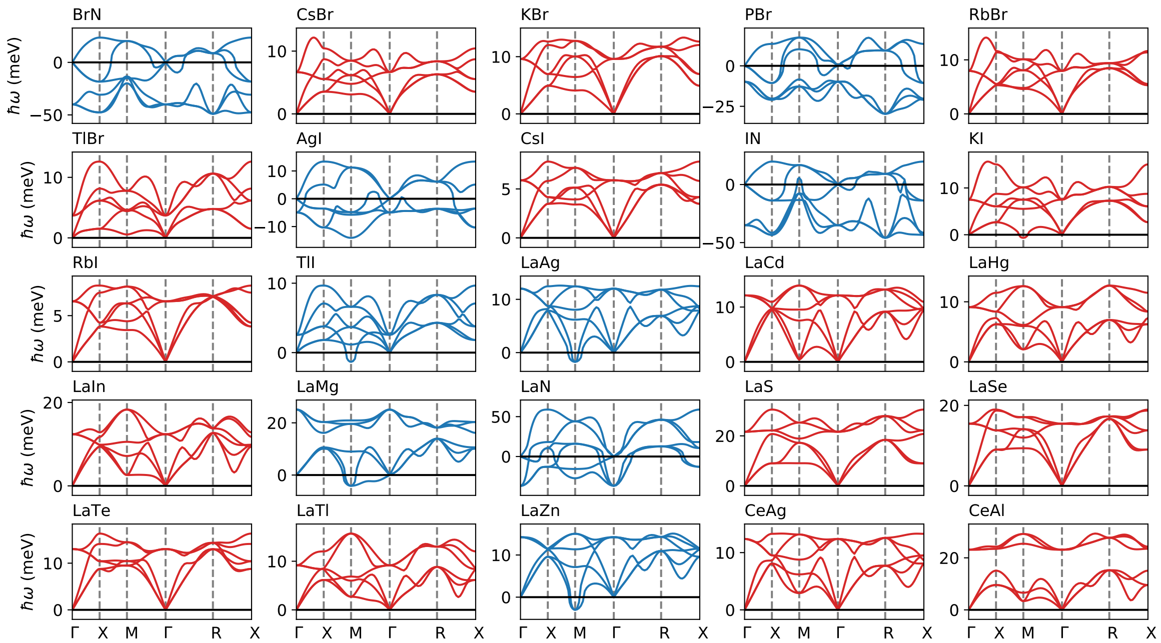

FIG. S1. *Continued.*

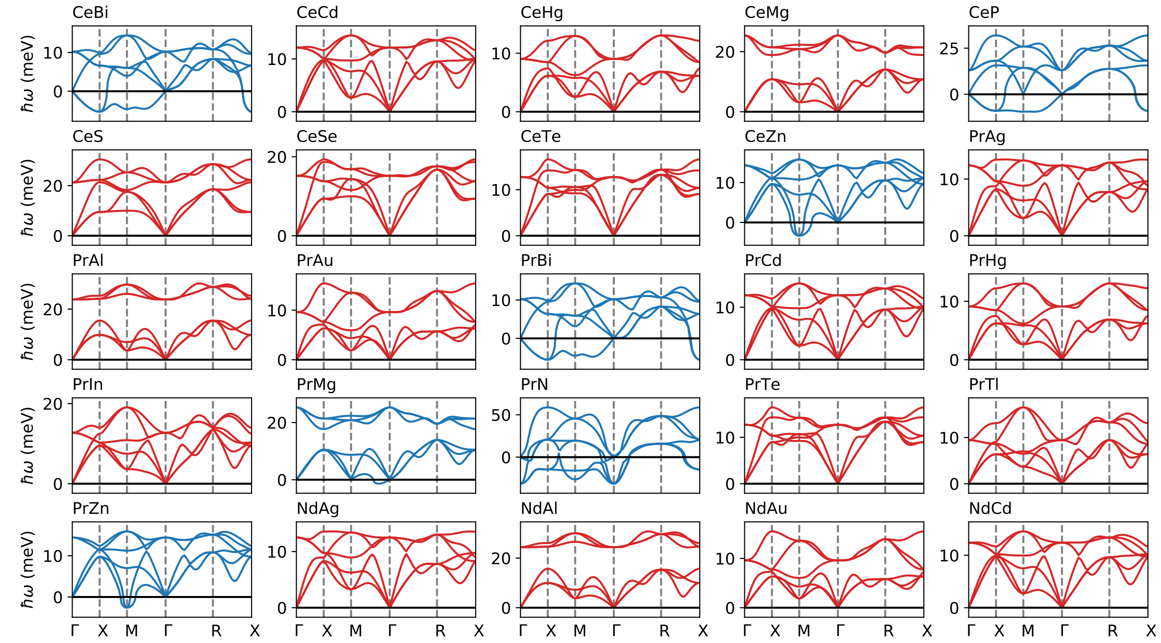

FIG. S1. *Continued.*

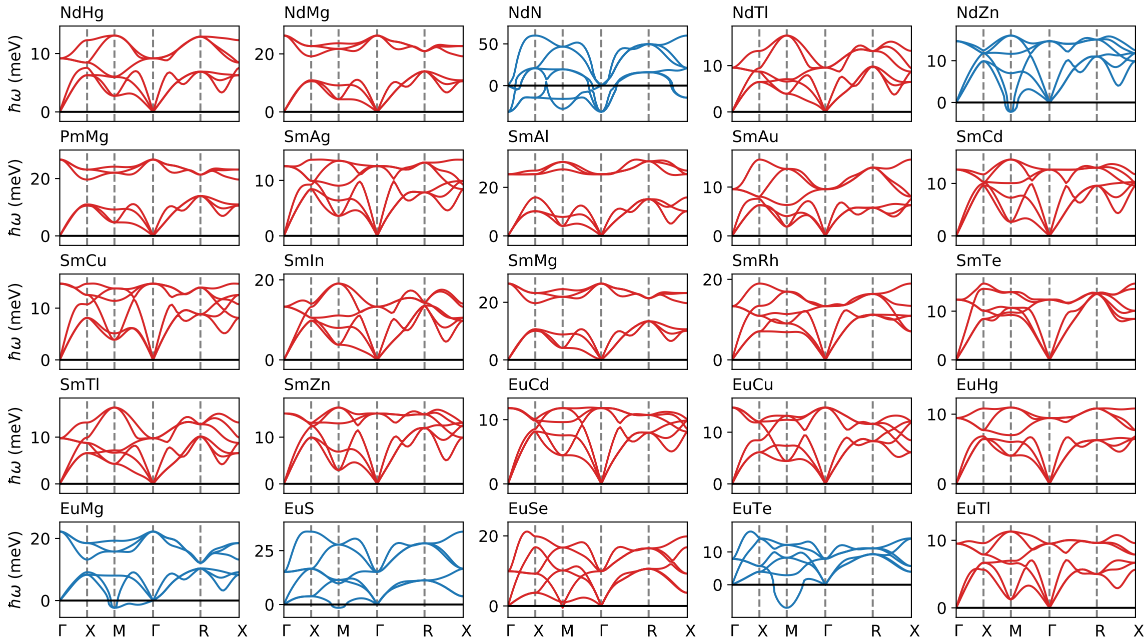

FIG. S1. *Continued.*

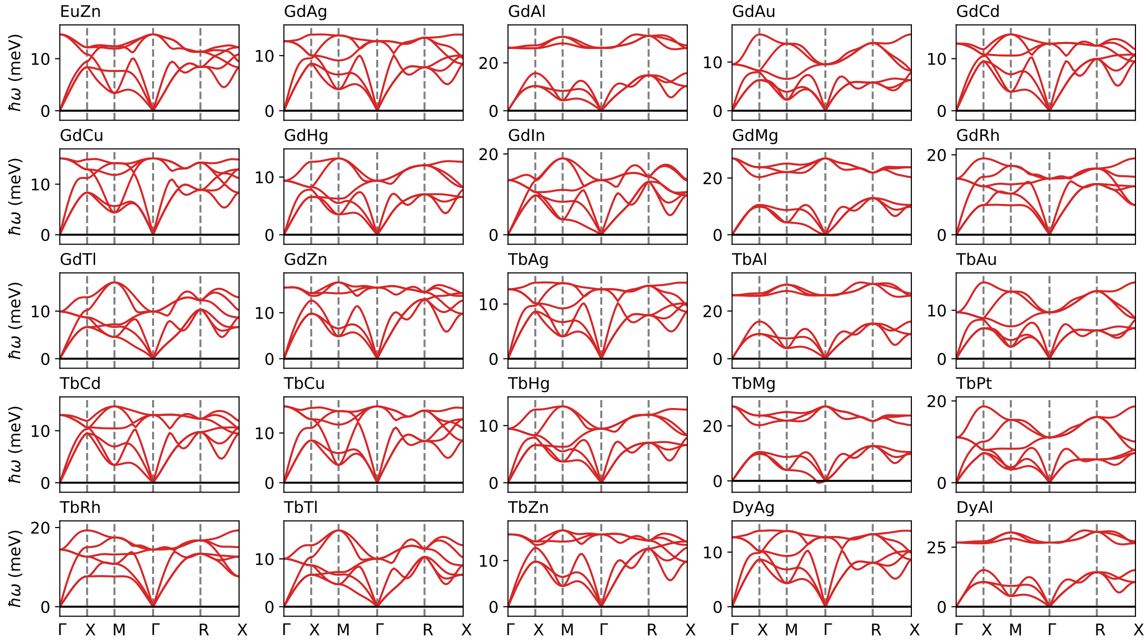

FIG. S1. *Continued.*

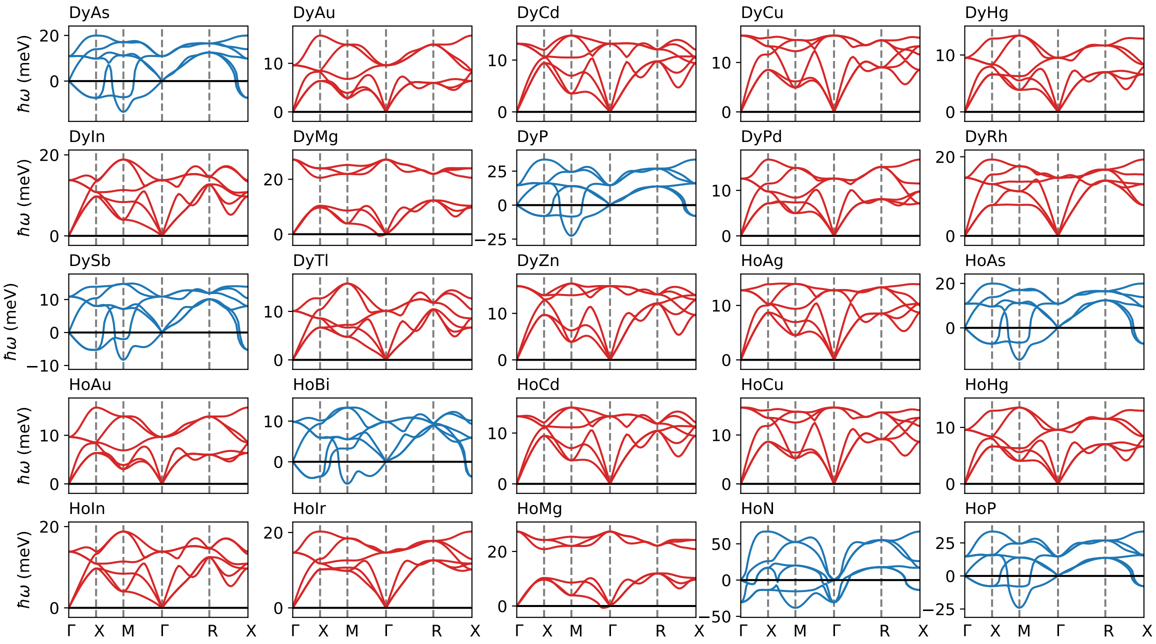

FIG. S1. *Continued.*

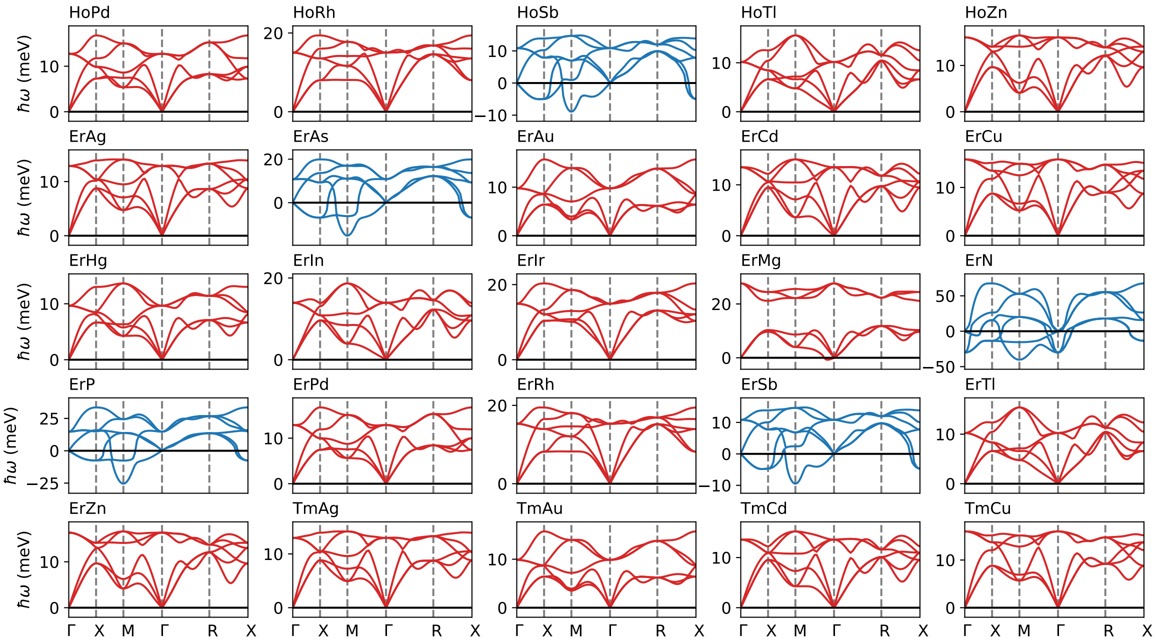

FIG. S1. *Continued.*

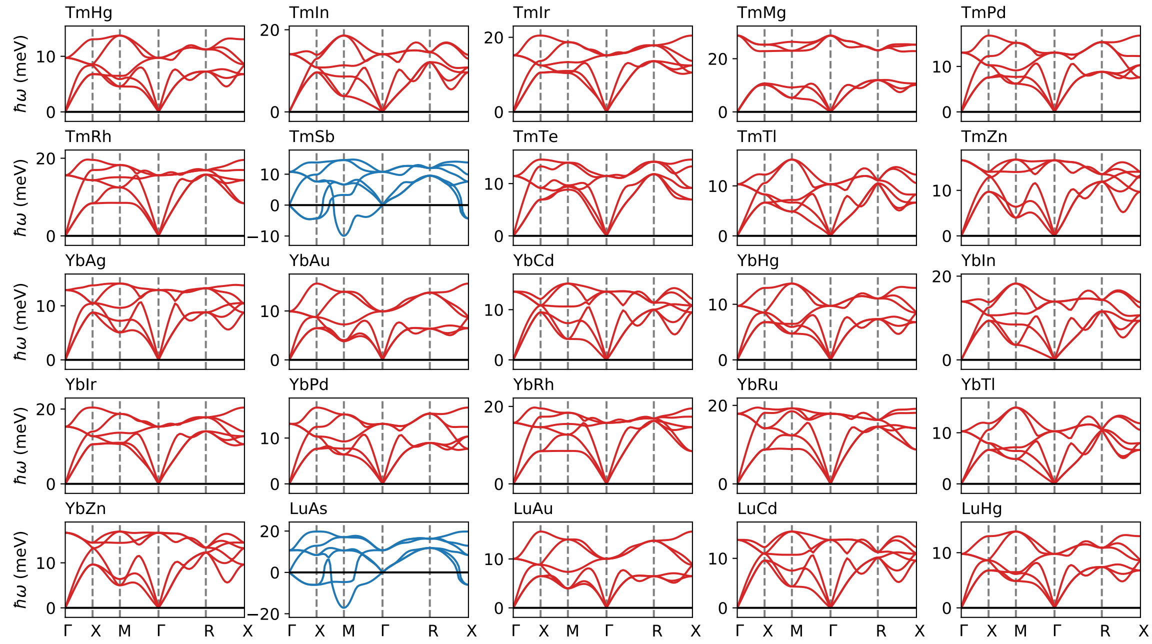

FIG. S1. *Continued.*

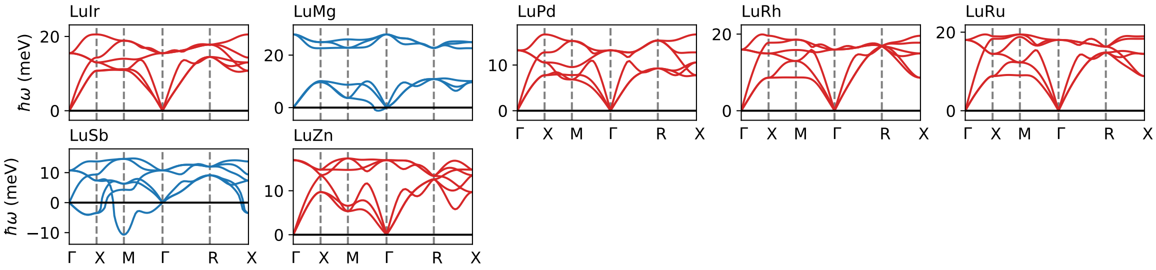

FIG. S1. *Continued.*

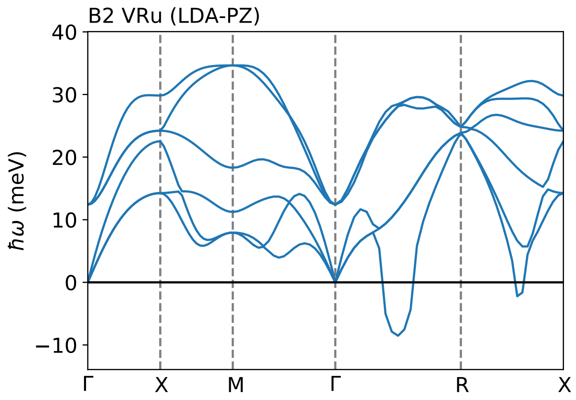

FIG. S2. The phonon dispersions for the VRu in the B2 structure (a = 2.948 Å) using the LDA-PZ functional. A 4×4×4 q grid is used.
